# Association of TyG Index and TG/HDL-C Ratio with Trajectories of Depressive Symptoms: Evidence from China Health and Retirement Longitudinal Study

**DOI:** 10.3390/nu16244300

**Published:** 2024-12-12

**Authors:** Tingting Guo, Qing Zou, Qi Wang, Yi Zhang, Xinyuan Zhong, Hantong Lin, Wenxuan Gong, Yingbo Wang, Kun Xie, Kunpeng Wu, Feng Chen, Wen Chen

**Affiliations:** 1 Department of Medical Statistics, School of Public Health, Sun Yat-sen University, 74 Zhongshan Second Rd, Guangzhou 510080, China; guott6@mail2.sysu.edu.cn (T.G.); zouq25@mail2.sysu.edu.cn (Q.Z.); wangq633@mail2.sysu.edu.cn (Q.W.); zhangy2553@mail2.sysu.edu.cn (Y.Z.); zhongxy85@mail2.sysu.edu.cn (X.Z.); linht35@mail2.sysu.edu.cn (H.L.); gongwx5@mail2.sysu.edu.cn (W.G.); wangyb68@mail2.sysu.edu.cn (Y.W.); xiek9@mail2.sysu.edu.cn (K.X.); wukp6@mail2.sysu.edu.cn (K.W.); 2 Department of Clinical Research, The Eighth Affiliated Hospital, Sun Yat-sen University, 3025 Shennan Zhong Rd, Shenzhen 518033, China; chenf329@mail.sysu.edu.cn; 3 Center for Migrant Health Policy, Sun Yat-sen University, 74 Zhongshan Second Rd, Guangzhou 510080, China

**Keywords:** trajectory, depressive symptoms, triglyceride–glucose (TyG) index, TG/HDL-C ratio, longitudinal study

## Abstract

Objectives: To explore whether the triglyceride–glucose (TyG) index and the triglyceride to high-density lipoprotein cholesterol (TG/HDL-C) ratio are associated with the trajectories of depressive symptoms. Methods: In this longitudinal study, 4215 participants aged 45 years and older were recruited from the China Health and Retirement Longitudinal Study from 2011 to 2018. The trajectories of depressive symptoms, measured by the 10-item Center for Epidemiologic Studies Depression Scale (CESD-10), were identified using group-based trajectory modeling. Multinomial logistic models and restricted cubic spline analysis were used to investigate the relationships between the TyG index and the TG/HDL-C ratio and the trajectories of depressive symptoms. Stratified analyses were conducted based on sex, age, place of residence, and body mass index (BMI). Results: Five distinct trajectories of depressive symptoms characterized by stable low, stable moderate, decreasing, increasing, and stable high were identified during a follow-up of 7 years. The associations of the TyG index and the TG/HDL-C ratio with trajectories of depressive symptoms are not entirely consistent. After adjusting for covariates, a higher TyG index at baseline was associated with lower odds of being on the decreasing trajectory of depressive symptoms (*OR*_ad_ = 0.61, 95% CI: 0.40–0.92) compared to the stable low trajectory, and restricted cubic spline analysis revealed a negative linear relationship between the TyG index and the likelihood of a decreasing trajectory of depressive symptoms. However, the relationship between the TG/HDL-C ratio and the decreasing trajectory of depressive symptoms was no longer statistically significant when all confounders were controlled (*OR*_ad_ = 0.72, 95% CI: 0.50−1.04). Additionally, this negative association between the TyG index and decreasing trajectory of depressive symptoms was observed among 45–64-year-old individuals, female participants, those living in rural areas, and those with a normal BMI. Limitations: This study was conducted in a middle-aged and elderly population in China, and extrapolation to other regions and populations requires further confirmation. Conclusions: Compared to the TG/HDL-C ratio, the TyG index may be a better predictor for trajectories of depressive symptoms in middle-aged and older adults. Considering that the pathology of depression progresses long term, our findings may have utility for identifying available and reliable markers for the development of depression.

## 1. Introduction

Depression (also known as depressive disorder) is a common mental disorder characterized by a persistent depressed mood and/or markedly diminished interest or pleasure in activities, with substantial economic costs [1]. Depression is a major contributor to the global burden of disease, and age-standardized disability-adjusted life years increased by 16.4% for depression between 2010 and 2021 [2]. A meta-analysis revealed that more than one-third of the world’s elderly population suffers from depression [3]. An estimated 20.0% of the elderly population in China experiences depression, and both lifetime and 12-month incidences of depression were more common in older age groups than in adults aged 18–34 years [4,5]. 

Many studies have demonstrated that diabetes increases the risk of depression, but the relationship between depression and diabetes is still not well understood [6,7,8]. Recent research suggests that the link between diabetes and depression is primarily mediated by insulin resistance (IR) [9,10,11]. Growing evidence indicates a link between IR and depression [12,13,14]. For example, a cohort study revealed that three indicators (a high triglyceride–high-density lipoprotein ratio, fasting plasma glucose, and waist circumference) for IR positively predicted the occurrence of major depression [15]. Moreover, systematic reviews and meta-analyses have similarly reported that IR increases the likelihood of depression [16,17].

For diagnosing IR, the hyperinsulinemic–euglycemic clamp is the gold standard. However, it is expensive, time-consuming, and labor-intensive, making it impractical for routine clinical use [18]. Previous studies have demonstrated that the triglyceride–glucose (TyG) index, which is based on fasting triglycerides (TGs) and fasting blood glucose (FBG), and the triglyceride to high-density lipoprotein cholesterol (TG/HDL-C) ratio are simple and credible surrogate markers for IR. These markers strongly correlate with the clamp results and are more suitable for clinical practice and large-scale epidemiological studies [19,20]. Many studies have suggested that individuals with a higher TyG index are more likely to experience depressive symptoms, but most of these studies were cross-sectional [21,22,23,24]. In addition, a recent systematic review involving 58,981 participants also demonstrated that an increase in the TyG index increased the risk of depression [25]. Zheng et al. found that a higher TyG index at baseline is related to the progression of depressive symptoms through a cohort study [26]. In addition, previous studies have indicated that elevated TG levels and decreased HDL-C levels are associated with depression [27,28,29]. Han’s cross-sectional study explored the association between the TG/HDL-C ratio and depression and found that this association was present only in men (*OR* = 1.041, *p* = 0.028) [30]. In a cohort study with a 9-year follow-up period, a higher TG/HDL-C ratio was positively associated with an increased incidence of major depressive disorder (*HR* = 1.89, 95% CI = 1.15, 3.11) [15]. However, the relationship between indicators of IR and the trajectories of depressive symptoms has never been investigated. Depressive symptoms fluctuate over time, and previous research has demonstrated the heterogeneity of depression trajectories in middle-aged and elderly people [31,32,33]. Considering that a growing body of research suggests that persistent and high levels of depressive symptom trajectories are positively associated with adverse health outcomes [34,35], it is worthwhile to identify risk factors for adverse depressive symptom trajectories to help formulate targeted strategies to prevent and intervene in depression.

Thus, to explore whether IR could impact the development of trajectories of depressive symptoms among middle-aged and older adults, we investigated the association between the indicators of IR (the TyG index and the TG/HDL-C ratio) and the trajectories of depressive symptoms using data from the China Health and Retirement Longitudinal Study.

## 2. Materials and Methods

### 2.1. Database and Study Population

This study utilized public data from the China Health and Retirement Longitudinal Study (CHARLS) [36], a nationally representative longitudinal survey focusing on the health and socioeconomic conditions of middle-aged and older Chinese individuals. The baseline survey was conducted in 2011–2012 and involved 17,708 adults from 10,257 households. Details on the CHARLS have previously been published [37,38]. Briefly, the baseline samples were selected through multistage probability sampling to ensure representativeness. It was first stratified by region, urbanization level, and economic development. There were 150 county-level units that were then randomly chosen with a probability-proportional-to-size sampling technique, resulting in a final sample of counties distributed across 28 provinces in China. Following the baseline survey, CHARLS respondents have been followed every two years, using a face-to-face computer-assisted personal interview. Physical measurements are made at every 2-year follow-up, and blood samples are collected once every two follow-up periods. The study received ethical approval from Peking University’s Institutional Review Board (No. IRB00001052-11014 and No. IRB00001052-11015), and all participants provided written informed consent.

The study participants were recruited from four waves in 2011, 2013, 2015, and 2018. In alignment with the research aims, we excluded participants based on the following criteria: (1) absence of data on depressive symptoms at baseline; (2) lack of fasting blood data such as TG, HDL-C, and glucose at baseline; (3) being younger than 45 years old or having an unrecorded age (because the CHARLS study focuses on individuals aged 45 and above); (4) having missing or abnormal values for baseline covariates which are described in the Assessment of Covariates subsection; (5) cannot be tracked in subsequent follow-ups. There were 7789 participants with complete data at baseline (in 2011). Among them, a total of 4215 participants who had complete measurements of depressive symptoms at each follow-up survey were enrolled in our study. The full process of participant selection is depicted in Supplementary Figure S1.

### 2.2. Assessment of Depressive Symptoms

Depressive symptoms were evaluated using the Chinese version of the 10-item Center for Epidemiologic Studies Depression Scale (CESD-10). It indicated adequate reliability (Cronbach α = 0.78–0.79 [39]) and validity (CFI = 0.99, WRMR = 0.80, RMSEA = 0.045 in the two-factor model [40]) for China’s community-dwelling older population. Participants rated their frequency of ten depressive symptoms in the past week on a 0-to-3 scale, from “rarely or none of the time” (<1 day) to “most or all of the time” (5–7 days). The final score ranges from 0 to 30, with higher scores indicating more severe symptoms, and a score of 10 or above is considered to indicate significant clinical depressive symptoms [41].

### 2.3. Assessment of Indicators of IR

Following a standard protocol, venous blood was obtained from each participant by the trained staff of the Chinese Center for Disease Control and Prevention (China CDC). FBG, TG, and HDL-C levels were measured using an enzymatic colorimetric test. The within-assay coefficients of variation for FBG, TG, and HDL-C were 0.90%, 1.50%, and 1.00%, respectively, while the between-assay coefficients of variation were 1.80%, 1.80%, and 1.30%, respectively. Comprehensive details regarding the collection, processing, transportation, storage, and analysis of the blood samples, as well as the quality control and external quality assessment for the laboratory, have been described in the CHALRS Blood Sample Users’ Guide [42]. The TyG index was computed using the formula Ln [fasting triglyceride (mg/dL) × fasting glucose (mg/dL)/2], and the TG/HDL-C ratio was calculated as triglyceride (mg/dL)/high-density lipoprotein cholesterol (mg/dL). Since there is no standard threshold for identifying IR [43], referring to previous studies [21,44,45,46], we employed a quartile-based categorization for the TyG index and the TG/HDL-C ratio.

### 2.4. Assessment of Covariates

Informed by previous epidemiological studies, we controlled for potential confounders collected at baseline. We gathered demographic data (age, gender, household income, marital status, educational level, and residence) [26,47,48], health behaviors (smoking status, drinking status, and sleep duration) [49,50,51], and baseline health conditions (health status, disabilities, hypertension, dyslipidemia, cardiovascular disease, diabetes, and cognition scores) [50,52,53,54]. To be concrete, marital status was defined as a dichotomous variable, with “Married” representing legally married or in a common-law relationship and “Single” involving never married, separated, widowed, and divorced. Educational level was divided into “Illiterate”, “Primary school and below”, or “Middle school and above”. Place of residence was identified as either “Rural” or “Urban”. Smoking habits were classified as “Never”, “Former”, or “Current”, while drinking frequency was categorized into “Never”, “<1 time/month”, or “≥1 time/month”. Based on self-reporting, health status was identified as “Good”, “Fair”, or “Poor”, and disability status was indicated as “No” or “Yes”. Medical histories such as hypertension, dyslipidemia, cardiovascular disease (CVD), and diabetes were validated through physician diagnoses and marked as “No” or “Yes”. Somatic–psychiatric comorbidity was defined as CESD-10 ≥ 10 with one or more somatic disorders of comorbid disability, hypertension, dyslipidemia, CVD, and diabetes. Overall cognitive function was assessed based on the total score of episodic memory and mental status on a scale from 0 to 21, with a higher score indicating superior cognitive function. Furthermore, we recorded clinical laboratory test data [55,56,57,58], including body mass index (BMI) measured by Seca™ 213 Monitor (China seca (Hangzhou) Co., Ltd., Hangzhou, China) and Omron™ HN-286 Monitor (Kerui Technology (Yangzhou) Co., Ltd., Yangzhou, China), systolic and diastolic blood pressure (calculated as the average of three independent measurements using Omron™ HEM-7112 Monitor, Omron (Dalian) Co., Ltd., Dalian, China), total cholesterol (TC), HDL-C, low-density lipoprotein cholesterol (LDL-C), glycated hemoglobin (HbA1c), and C-reactive protein (CRP). BMI and blood pressure were evaluated through physical examination, and the remaining tests were conducted using an overnight fasting blood sample.

### 2.5. Statistical Analysis

Continuous variables were presented as the means and standard deviations (SDs) or medians and interquartile ranges (IQRs), and categorical variables were presented as counts and percentages. Baseline characteristics were compared between participants with different depressive symptom trajectories by analysis of variance (ANOVA) for continuous variables and the chi-square test for categorical variables. Using group-based trajectory modeling (GBTM), we constructed a censored normal distribution model using the Stata traj plugin [59] to evaluate the trajectories of depressive symptoms across four visits. The optimal number and shape of trajectories were determined by three criteria [60,61]: (1) the minimum absolute value of the Bayesian information criterion (BIC), (2) the average posterior probability of assignment (APPA) (>0.7 was considered acceptable) among each trajectory, and (3) ensuring that the size of each subgroup is not less than 5%. Following a comprehensive analysis, a five-trajectory model was found to best meet the established criteria (Supplementary Table S1).

We utilized multinomial logistic regression to evaluate the association between the indicators of IR (the TyG index and the TG/HDL-C ratio) and the trajectories of depressive symptoms, and the results were reported as odds ratios (*OR*s) and 95% confidence intervals (95% CIs). Initially, covariate multicollinearity was examined using the tolerance and variance inflation factor (VIF), leading to the exclusion of “TC” (Supplementary Table S2). For confounder adjustment, five models were evaluated. Model 1 had no covariates, Model 2 adjusted for age and sex, Model 3 further included demographics and lifestyle behaviors, Model 4 added baseline health conditions, and Model 5 incorporated clinical laboratory indicators. To further understand this relationship, we conducted a trend test on the quartiles of the indicators of IR. Additionally, we analyzed the dose–response association between the baseline TyG index and depressive symptom trajectories using a restricted cubic spline (RCS) regression model. For the TG/HDL-C ratio, we did not conduct the RCS analysis since it was not significantly associated with depressive symptom trajectories under Model 5. Subgroup analyses were further performed to examine potential effect modifications stratified by age (45–64 or ≥65 years), sex (male or female), place of residence (rural or urban), and BMI (<24 or ≥24). We chose these stratifications according to previous research [4,55,62]. In the sensitivity analysis, we excluded participants who had treatments for diabetes (taking Chinese traditional medicine, taking Western modern medicine, or taking insulin injections) or depression (receiving psychiatric or psychological treatment, taking antidepressants, taking tranquilizers or sleeping pills) [63,64].

Stata 15.0 (Stata Corp LLC, College Station, TX, USA) and R 4.2.3 (www.r-project.org) were used for all analyses, and a two-sided *p* value < 0.05 was considered to indicate statistical significance.

## 3. Results

### 3.1. Trajectories of Depressive Symptoms in Overall Population

**Figure 1:**
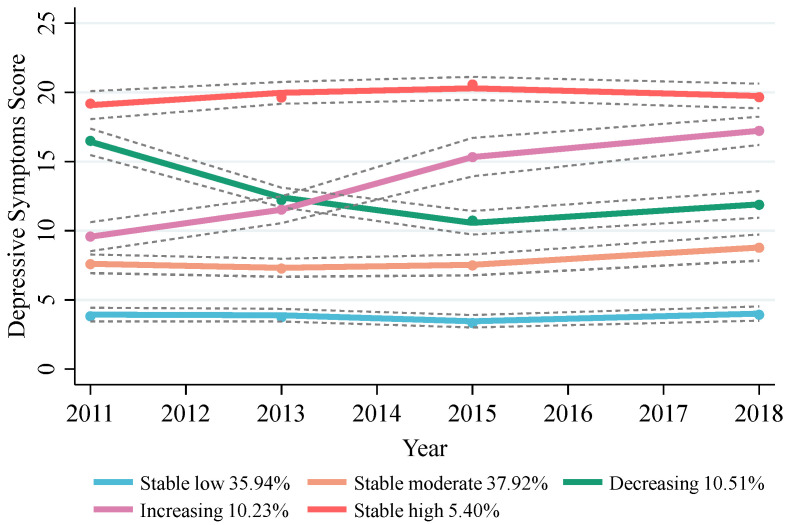
The trajectories of depressive symptoms in the study participants from 2011 to 2018. The trajectories are shown as solid lines, and the 95% confidence intervals (CIs) are shown as the dashed line.

This study included a total of 4215 participants (2017 males and 2198 females) with a mean age of 57.27 years (SD = 7.80 years). Throughout the 7-year follow-up, five distinct trajectories of depressive symptoms were identified (Figure 1). The “stable low” trajectory included 1510 participants (35.94%) who consistently exhibited low CESD-10 scores, with mean scores ranging from 3.00 (SD = 2.60) [median: 3, IQR: 1–4] to 3.53 (SD = 2.97) [median: 3, IQR: 1–5]. The “stable moderate” trajectory involved 1667 participants (37.92%) who maintained moderate CESD-10 scores, with mean scores between 7.48 (SD = 3.83) [median: 7, IQR: 5–10] and 9.12 (SD = 4.42) [median: 9, IQR: 6–12]. The “decreasing” trajectory comprising 415 participants (10.51%) began with moderately high scores (mean = 17.66, SD = 3.89) [median: 17, IQR: 15–20] and eventually decreased to lower scores (mean = 10.80, SD = 4.55) [median: 11, IQR: 8–14]. The “increasing” trajectory, seen in 399 participants (10.23%), started with moderate scores (mean score = 9.36, SD = 4.19) [median: 10, IQR: 6–12] and subsequently escalated to high scores (mean = 18.06, SD = 4.67) [median: 18, IQR: 15–21]. Finally, the “stable high” trajectory included 224 participants (5.40%) who consistently maintained high scores, with mean scores ranging from 19.34 (SD = 4.19) [median: 19, IQR: 15–23] to 20.80 (SD = 4.89) [median: 21, IQR: 17–25]. Additional details on the CESD-10 scores across the five trajectories in the four waves are provided in Supplementary Figure S2.

### 3.2. Baseline Characteristics of Participants with Different Depressive Symptom Trajectories

Table 1 provides an overview of the baseline characteristics of the study participants with different depressive symptom trajectories. Compared to participants with a “stable low” trajectory, participants with other trajectories were generally older, more likely to be female, had lower household incomes and education levels, resided in rural areas, and had a higher likelihood of residing in rural areas and being single.

**Figure 2:**
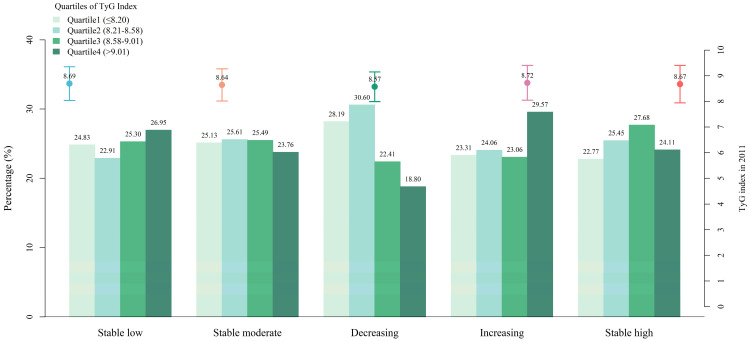
Descriptions of the baseline TyG index based on the trajectories of depressive symptoms. The dot and error bar represent the mean and standard deviation of the baseline triglyceride–glucose (TyG) index in the five trajectory groups, respectively.

**Figure 3:**
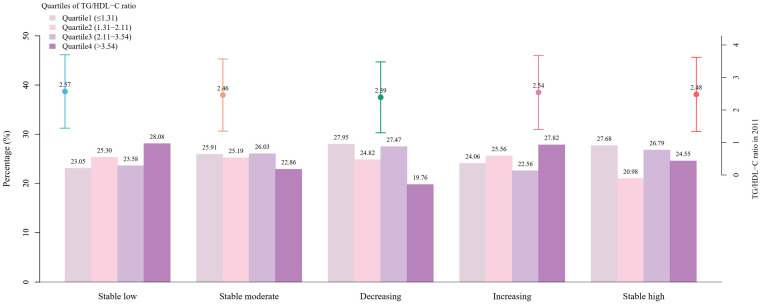
Descriptions of the baseline TG/HDL-C ratio based on the trajectories of depressive symptoms. The dot and error bar represent the mean and standard deviation of the baseline triglyceride to high-density lipoprotein cholesterol (TG/HDL-C) ratio in the five trajectory groups, respectively.

At baseline, the overall mean TyG index and TG/HDL-C ratio were 8.66 (SD = 0.65) and 2.50 (SD = 1.12), respectively. Significant differences were observed in the average levels of the baseline TyG index and TG/HDL-C ratio across the five trajectories (*p* < 0.05), with the “decreasing” trajectory presenting the lowest TyG index (mean = 8.57, SD = 0.58) and the lowest TG/HDL-C ratio (mean = 2.39, SD = 1.09). After classifying the TyG index and the TG/HDL-C ratio into quartiles, the proportion in each quartile was depicted, as shown in Figure 2 and Figure 3. It was noted that for the “decreasing” trajectory, the proportion of participants in the highest quartile (Q4) of the TyG index and the TG/HDL-C ratio was the lowest, accounting for only 18.80% and 19.76%, respectively.

### 3.3. The Association Between the Indicators of IR and the Trajectory of Depressive Symptoms

**Figure 4:**
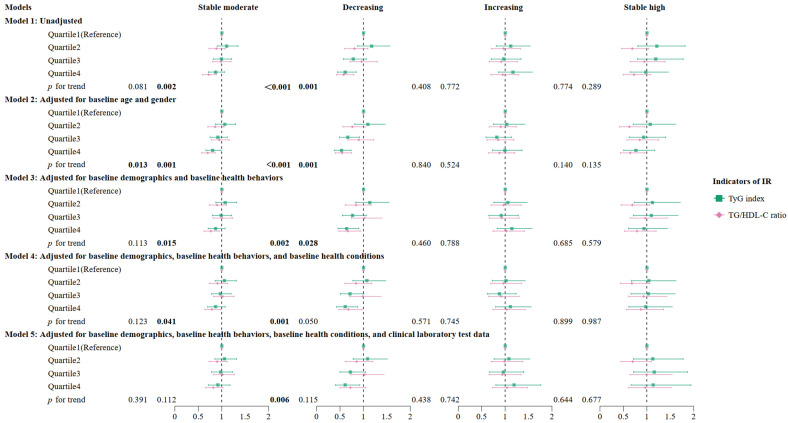
The association between quartiles of the TyG index and the TG/HDL-C ratio and trajectories of depressive symptoms. Abbreviations: *OR*, odds ratio; CI, confidence interval; TyG, triglyceride–glucose; TG/HDL-C, triglyceride to high-density lipoprotein cholesterol. The demographic factors included age, sex, household income, marital status, educational level, and residence. Health behaviors consisted of smoking status, drinking status, and hours of sleep. Baseline health conditions included health status, disabilities, hypertension, dyslipidemia, cardiovascular disease (CVD), diabetes, and cognition scores. Clinical laboratory test data consisted of body mass index (BMI), systolic blood pressure, diastolic blood pressure, low-density lipoprotein cholesterol (LDL-C), glycated hemoglobin, C-reactive protein (CRP), and high-density lipoprotein cholesterol (HDL-C, only for analyzing the TyG index).

The multinomial logistic regression examined the association between the quartiles of the TyG index and the TG/HDL-C ratio and the trajectories of depressive symptoms (Figure 3 and Figure 4). For the TyG index, taking the “stable low” trajectory as the reference group, the unadjusted *OR* (95% CI) for participants in the fourth quartile (Q4) on a “decreasing” trajectory compared to the first quartile (Q1) was 0.61 (0.45–0.85). Similarly, for the TG/HDL-C ratio, the unadjusted *OR* (95% CI) for participants in Q4 on the “decreasing” trajectory compared to Q1 was 0.58 (0.42–0.80). Additionally, for the “stable moderate” trajectory, participants in Q4 had an unadjusted *OR* (95% CI) of 0.72 (0.59–0.88) compared to those in Q1.

**Figure 5:**
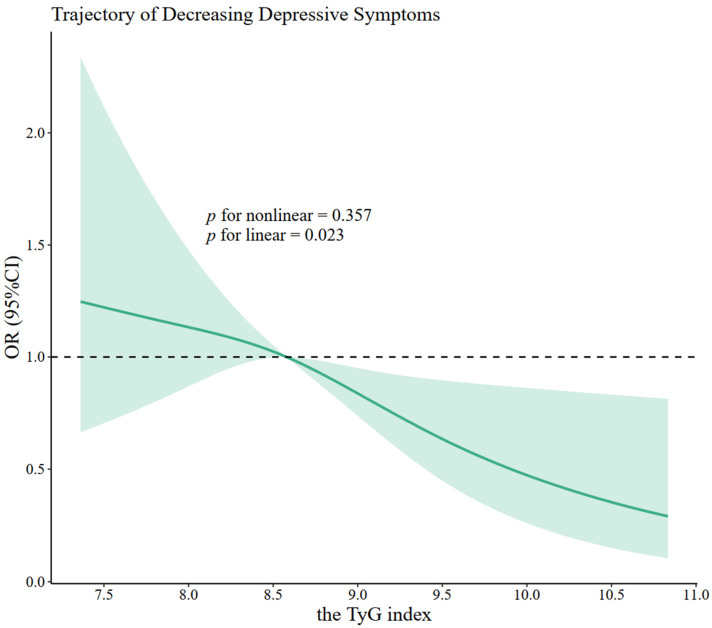
The restricted cubic spline curve for the association of the TyG index with the trajectory of decreasing depressive symptoms.

After further adjustments for covariates in Models 2–5, individuals in the highest quartile of the TyG index were still significantly associated with the “decreasing” trajectory. The *OR*s (95% CIs) were 0.53 (0.38–0.73), 0.64 (0.46–0.90), 0.61 (0.43–0.88), and 0.61 (0.40–0.92) under Models 2–5, respectively. No significant associations between the TyG index and depressive symptoms were found in the other trajectory groups. Furthermore, the results of the trend test indicated that with an increase in the TyG index quartile, there was a gradual decrease in the *OR*s (95% CIs) of the “decreasing” trajectory (*p* for trend < 0.05). The restricted cubic spline regression models also showed linear associations (*p* = 0.023) between the baseline TyG index and the “decreasing” trajectory (Figure 5).

The highest quartile of the TG/HDL-C ratio was also significantly associated with the “decreasing” trajectory under Models 2–4, and the *OR*s (95% CIs) were 0.54 (0.40–0.75), 0.67 (0.48–0.93), and 0.68 (0.48–0.97), respectively. Additionally, significant associations were found between the TG/HDL-C ratio and the “stable moderate” trajectory under Models 2–4, with *OR*s (95% CIs) of 0.70 (0.58–0.86), 0.77 (0.62–0.94), and 0.79 (0.64–0.98), respectively. The trend test results indicated that with an increase in the TG/HDL-C ratio quartile, there was a gradual decrease in the *OR*s (95% CIs) of the “decreasing” trajectory for Models 1–3 (*p* for trend < 0.05) and the “stable moderate” trajectory for Models 1–4 (*p* for trend < 0.05).

The results of the subgroup analysis are displayed in Supplementary Figures S3 and S4. The highest quartile of the TyG index was associated with a lower likelihood of the “decreasing” trajectory among females (*OR* = 0.52, 95% CI: 0.29–0.93), individuals aged 45–64 years (*OR* = 0.54, 95% CI: 0.34–0.86), rural residents (*OR* = 0.57, 95% CI: 0.35–0.93), and those with a BMI under 24 (*OR* = 0.54, 95% CI: 0.30–0.98). The subgroup of “65 years and older” displayed another interesting result, with individuals in the highest TyG index quartile being more likely to belong to the “increasing” trajectory group (*OR* = 3.53, 95% CI: 1.34–9.29). Regarding the TG/HDL-C ratio, the highest quartile group was associated with a lower likelihood of the “decreasing” trajectory (*OR* = 0.60, 95% CI: 0.37–0.99) and the “stable moderate” group (*OR* = 0.71, 95% CI: 0.51–0.99) among females. According to the sensitivity analysis, after excluding participants who had been treated for diabetes or depression, the association between the TyG index and these trajectories remained essentially unchanged, suggesting the robustness of the main analysis results (Supplementary Table S3). However, the association between the TG/HDL-C ratio and “decreasing” and “stable moderate” trajectories was statistically significant only in Models 1–3. (Supplementary Table S4).

The green line represents the odds ratio (*OR*), and the shaded area represents the 95% confidence interval (CI). The restricted cubic spline regression analysis was performed by using Model 5 in Figure 4 (adjusted for baseline demographics, baseline health behaviors, baseline health conditions, and clinical laboratory test data) and fitted with 3 knots at the 5th, 50th, and 95th percentiles of the baseline TyG index.

## 4. Discussion

In this national cohort study, instead of simply categorizing depression into normal and depressed groups, the GBTM model was used to gain a more detailed understanding of the longitudinal changes in depression in individuals over time. We identified five distinct trajectories of depressive symptoms during a 7-year follow-up period: stable low, stable moderate, decreasing, increasing, and stable high. Individuals in the highest quartile of the TyG index were associated with a lower likelihood of being on the decreasing trajectory of depressive symptoms than those in the lowest TyG index quartile. The association between the TG/HDL-C ratio and trajectories of depressive symptoms was statistically significant when the demographic factors, health behaviors, and baseline health conditions were adjusted. However, the association was no longer statistically significant when clinical laboratory test data were controlled for. To our knowledge, this is the first study to reveal the relationships between the TyG index and the TG/HDL-C ratio and trajectories of depressive symptoms among middle-aged and older people.

The finding on trajectories of depressive symptoms is consistent with previous findings from China and other countries that support heterogeneous depression trajectories [31,47]. For example, four trajectories of depressive symptoms (persistently low, increasing, declining, and persistently high) were identified among 7573 older adults from the National Health and Aging Trends Study [32]. In China, five depressive symptom trajectory groups (constantly low, constantly medium, increasing, decreasing, and constantly high) were identified among 9264 middle-aged and older adults from the CHARLS [33]. Similarly, five distinct trajectories of depressive symptoms (low, decreasing, remitting, increasing, and high) were also observed in the Netherlands [35]. Differences in the shape and number of depression trajectories might be due to different research designs as well as the follow-up time.

Previously, only a few longitudinal studies explored the association between the TyG index and depression. Existing evidence based on the CHARLS suggested that a high TyG index at baseline exacerbated the development of depression [26], and the TyG index was shown to weakly predict depressive symptoms in women aged 45 years and older [65]. In addition, a cohort study with more than 200,000 participants revealed that elevated triglyceride and glucose levels heightened the risk of depression [66]. Our findings revealed that the highest TyG index quartile was significantly negatively associated with a decreasing trajectory of depressive symptoms. Moreover, the RCS regression models also showed a negative linear relationship between the TyG index and the likelihood of a decreasing trajectory of depressive symptoms. Meanwhile, subgroup analyses revealed that individuals aged 65 years and older with a high TyG index were at increased risk of having increasing depressive symptom trajectories. IR develops with age [67,68], consequently elevating the risk of metabolic disorders [69]. Since the TyG index is a reliable surrogate for IR, our findings suggest that health interventions to lower IR, such as a healthy diet [70,71] and increased exercise [68,72], may have a dual effect on reducing the risk of metabolic disease and depression. It is worth noting that the CHARLS-based study showed that the prevalence of physical inactivity among China’s middle-aged and elderly population was as high as 20.64% [73], and sustained light physical activity could alleviate individual depressive symptoms [74], highlighting the need for enhanced health education within this demographic.

There are limited studies on the correlation between the TG/HDL-C ratio and depression, with only one cohort study examining the longitudinal relationship between the TG/HDL-C ratio and depression [15,30,75]. Watson KT et al. [15] found that a high TG/HDL-C ratio predicted major depression onset in individuals with elevated FBG. A case–control study from India reported that the positive association between the TG/HDL-C ratio and depression was observed exclusively in patients with type 2 diabetes mellitus [75]. One of the possible reasons why the association of the bTG/HDL-C ratio with depressive symptom trajectory was not statistically significant after controlling for all covariates was that most participants had normal FBG levels in our study. Notably, the mean FBG level in the decreasing trajectory group was the lowest among all trajectory groups (104.71 ± 25.21 mg/dL). The results of the association of the TyG index and the TG/HDL-C ratio with trajectories of depressive symptoms in the present study were not entirely consistent, with more robust results for the TyG index. This inconsistency may be attributed to differences in their correlation with IR. Although both the TyG index and the TG/HDL-C ratio are alternative indicators for assessing IR clinically [76], many studies have shown that the TyG index is a better biomarker for early identification of individuals with IR when compared with the TG/HDL-C ratio [77,78]. One possible explanation for this is that the key mechanisms of IR development include glucotoxicity and lipotoxicity [68], and the TyG index, a composite index that combines fasting glucose and lipids, may better represent IR. Furthermore, racial differences exist in the correlation between the TG/HDL-C ratio and IR, with the TG/HDL-C ratio being more accurate in predicting IR among Caucasians, Asians, and Hispanics over African Americans [20]. The results of this study suggest that the TyG index may be a better predictor of depression than the TG/HDL-C ratio, and more research is needed on the association of other indicators for IR with depression in the future.

In the present study, the link between IR and the trajectory of decreasing depressive symptoms may be explained by the following mechanisms. First, oxidative stress and inflammation play important roles in the development of IR [79]. IR also induces systemic low-grade inflammation [80], and oxidative stress and inflammation promote the occurrence of depression [81,82]. Second, the dysfunction of the hypothalamic–pituitary–adrenal (HPA) axis has been recognized as a key player in depression. The main reason for the increase in HPA axis activity is exposure to high levels of corticotropin-releasing hormone, adrenocorticotropic hormone, and glucocorticoids [82]. When IR occurs, it leads to the insensitivity of glucocorticoid receptors, resulting in excessive glucocorticoid levels [11], while IR is associated with dysfunction of the HPA axis [83,84]. This result supports the HPA axis dysfunction hypothesis of depression. Moreover, a recently published meta-analysis showed that N-acetyl aspartate (NAA) concentrations were significantly decreased in patients with chronic depression [85]. NAA is a biomarker of neuronal metabolic health, and neuroplasticity defects and hypometabolism are involved in the occurrence and development of depression [82]. Lee S et al. found that individuals with IR have lower NAA levels in the prefrontal lobe, and IR will reduce the vitality of brain neurons [86]. Peripheral insulin resistance leads to brain insulin resistance [87], and brain insulin resistance can cause neuroplasticity defects and lead to an increased risk of depression [88], suggesting that IR contributes to the persistence of depression due to the presence of neuronal plasticity defects and decreased brain metabolism. Additionally, there is increasing evidence that IR can affect dopamine signaling and that dopamine dysfunction is a major underlying mechanism of depression [82,89]. Furthermore, insulin resistance-related syndromes, such as diabetes and dementia, are well-known risk factors for depression [8,90]. In turn, depression may worsen IR [91], long-term depressive symptoms increase individuals’ risk of insulin resistance-related disorders [91,92,93,94], and the coexistence of depression and insulin resistance-related disorders can interact to jointly affect this risk. However, because follow-up IR indicator information was not collected in the CHARLS, an inverse causality between IR and depressive symptoms could not be verified.

In addition, consistent with He’s and Zhang’s findings that a significant positive association between IR and depression was discovered in females but not in males [65,95], the current subgroup analyses also suggested that the TyG index and the TG/HDL-C ratio are more reliable indicators of depression in females, possibly because women have higher levels of depression and are more likely to suffer from IR than men [96,97]. According to the extended Social Signal Transduction Theory of Depression proposed by Slavich GM and Sacher J, fluctuations in ovarian hormones influence women’s vulnerability to stress, altered brain structure and function, and inflammatory activity and reactivity, thereby increasing the risk of depression in women [98]. This partially explains the gender differences observed in the associations found in this study. The relationship between the TyG index and the trajectory of decreasing depressive symptoms was not significant in overweight participants, which supports the hypothesis of “jolly fat” for middle-aged and older adults in China [99]. However, studies in the United States have found that the positive association between the TyG index and depression is more significant in overweight and obese individuals [21]. This discrepancy may be partly due to cultural differences, as attitudes toward obesity vary by region. In traditional Chinese culture, weight gain in middle age is considered a sign of good luck. Being overweight or obese is related to increased wealth and a higher social status, rather than being a symbol of poor health. This may explain why the present study did not find an association between the TyG index and the trajectory of depression in overweight middle-aged and elderly populations. More research is needed to explore this difference in the future. Furthermore, a large body of research reports that middle-aged and older adults living in rural areas are more likely to suffer from depression than urban residents in China [62,100,101], and health literacy and the utilization of health services among rural residents are lower than among urban residents, which may result in the physical and mental health of rural residents being less susceptible to improvement [102,103,104].

This study has several limitations. First, scores on the CESD-10 were self-reported by the study participants, which might have been influenced by recall bias. Second, individuals with severe depression may have withdrawn from the study during the follow-up (Supplementary Table S5), and the number of people in the groups of trajectories of decreasing and stable high depressive symptoms may have been underestimated. Additionally, in this study, only data from individuals with complete information were included, which could result in selection bias. Third, the gold standard for assessing IR [105], the hyperinsulinemic–euglycemic clamp test, was unavailable in this study, so an individual’s true IR could not be realistically measured. However, it is much easier to test the TyG index and the TG/HDL-C ratio in practice. Fourth, this study was conducted in a middle-aged and older Chinese population, and it is uncertain whether these findings can be extrapolated to other regions and populations. Finally, although we controlled for relevant confounders in our analyses, residual bias was still possible in this study and could have affected the results. For example, dietary structure has not been investigated in the CHARLS, but studies have found that dietary structure affects an individual’s IR and depressive symptoms in China [106,107]. For instance, the salt-preserved vegetable–garlic dietary pattern was associated with an increased risk of depression, whereas the protective effect of the vegetable–egg–beans–milk dietary pattern was confirmed in a longitudinal study in Chinese older adults [107].

## 5. Conclusions

In summary, our study identified five trajectories of depressive symptoms among Chinese adults aged 45 years and older. The TyG index demonstrates a more robust association with the trajectory of depressive symptoms than the TG/HDL-C ratio. Compared to individuals with a lower TyG index at baseline, participants with higher TyG index levels have a lower likelihood of being on a decreasing trajectory of depressive symptoms. The negative associations were robust in the following subgroups: females, individuals aged 45–64 years, individuals living in rural areas, and individuals with a BMI less than 24. Considering that the TyG index is a reliable surrogate indicator of IR, the findings of this study not only support that the TyG index may be a potential biomarker for the occurrence and development of depression but also provide a possible research perspective on the biological mechanism of metabolic diseases and depression comorbidities. In addition, attention should be given to health interventions, such as healthy dietary patterns and exercise, from the perspective of the metabolic and mental health of middle-aged and elderly populations to promote healthy aging.

## Figures and Tables

**Table 1:** Baseline characteristics of study participants based on trajectories of depressive symptoms.

Characteristics	Total (*n* = 4215)	Stable Low (*n* = 1510)	Stable Moderate (*n* = 1667)	Decreasing (*n* = 415)	Increasing (*n* = 399)	Stable High (*n* = 224)	*p*
Age (years), Mean (SD)	57.27 (7.80)	56.71 (7.78)	57.46 (7.78)	57.69 (7.87)	57.78 (8.09)	57.85 (7.28)	0.012
Gender, *n* (%)							<0.001
Male	2017 (47.85)	900 (59.60)	779 (46.73)	153 (36.87)	134 (33.58)	51 (22.77)	
Female	2198 (52.15)	610 (40.40)	888 (53.27)	262 (63.13)	265 (66.42)	173 (77.23)	
Household income (RMB), Median (IQR)	10,800 (1440, 28,400)	15,750 (2518, 36,000)	10,100 (1480, 26,600)	5850 (1051, 20,000)	6380 (1150, 24,210)	4150 (1000, 14,800)	<0.001
Marital status, *n* (%)							<0.001
Married ^a^	3890 (92.29)	1427 (94.50)	1548 (92.86)	361 (86.99)	363 (90.98)	191 (85.27)	
Single ^b^	325 (7.71)	83 (5.50)	119 (7.14)	54 (13.01)	36 (9.02)	33 (14.73)	
Educational level, *n* (%)							<0.001
Illiterate	889 (21.09)	219 (14.50)	355 (21.30)	125 (30.12)	110 (27.57)	80 (35.71)	
Primary school and below	1819 (43.08)	577 (38.21)	746 (44.75)	191 (46.02)	190 (47.62)	112 (50.00)	
Middle school and above	1510 (35.82)	714 (47.28)	566 (33.95)	99 (23.86)	99 (24.81)	32 (14.29)	
Place of residence, *n* (%)							<0.001
Rural	2730 (64.77)	834 (55.23)	1108 (66.47)	324 (78.07)	295 (73.93)	169 (75.45)	
Urban	1485 (35.23)	676 (44.77)	559 (33.53)	91 (21.93)	104 (26.07)	55 (24.55)	
Smoking status, *n* (%)							<0.001
Never	2565 (60.85)	837 (55.43)	1015 (60.89)	272 (65.54)	271 (67.92)	170 (75.89)	
Former	354 (8.40)	138 (9.14)	147 (8.82)	31 (7.47)	23 (5.76)	15 (6.70)	
Current	1296 (30.75)	535 (35.43)	505 (30.29)	112 (26.99)	105 (26.32)	39 (17.41)	
Drinking status, *n* (%)							<0.001
Never	2741 (65.03)	886 (58.68)	1104 (66.23)	294 (70.84)	292 (73.18)	165 (73.66)	
<1 time/month	352 (8.35)	139 (9.21)	129 (7.74)	29 (6.99)	35 (8.77)	20 (8.93)	
≥1 time/month	1122 (26.62)	485 (32.12)	434 (26.03)	92 (22.17)	72 (18.05)	39 (17.41)	
Sleep duration (hours/night), Mean (SD)	6.43 (1.79)	6.95 (1.44)	6.38 (1.77)	5.60 (2.00)	6.08 (1.96)	5.42 (2.06)	<0.001
Health status, *n* (%)							<0.001
Good	1039 (24.65)	567 (37.55)	345 (20.70)	43 (10.36)	76 (19.05)	8 (3.57)	
Fair	2114 (50.15)	784 (51.92)	897 (53.81)	171 (41.20)	178 (44.61)	84 (37.50)	
Poor	1062 (25.20)	159 (10.53)	425 (25.49)	201 (48.43)	145 (36.34)	132 (58.93)	
Disabilities, *n* (%)							<0.001
No	3631 (86.14)	1348 (89.27)	1463 (87.76)	324 (78.07)	321 (80.45)	175 (78.13)	
Yes	584 (13.86)	162 (10.73)	204 (12.24)	91 (21.93)	78 (19.55)	49 (21.88)	
Hypertension, *n* (%)							0.002
No	3195 (75.80)	1192 (78.94)	1251 (75.04)	293 (70.60)	290 (72.68)	169 (75.45)	
Yes	1020 (24.20)	318 (21.06)	416 (24.96)	122 (29.40)	109 (27.32)	55 (24.55)	
Dyslipidemia, *n* (%)							0.686
No	3793 (89.99)	1349 (89.34)	1512 (90.70)	376 (90.60)	357 (89.47)	199 (88.84)	
Yes	422 (10.01)	161 (10.66)	155 (9.30)	39 (9.40)	42 (10.53)	25 (11.16)	
CVD, *n* (%)							<0.001
No	3717 (88.19)	1380 (91.39)	1477 (88.60)	334 (80.48)	351 (87.97)	175 (78.13)	
Yes	498 (11.81)	130 (8.61)	190 (11.40)	81 (19.52)	48 (12.03)	49 (21.88)	
Diabetes, *n* (%)							0.134
No	3969 (94.16)	1438 (95.23)	1560 (93.58)	387 (93.25)	370 (92.73)	214 (95.54)	
Yes	246 (5.84)	72 (4.77)	107 (6.42)	28 (6.75)	29 (7.27)	10 (4.46)	
Somatic–psychiatric comorbidity, *n* (%)							<0.001
No	3472 (82.37)	1483 (98.21)	1402 (84.10)	191 (46.02)	289 (72.43)	107 (47.77)	
Yes	743 (17.63)	27 (1.79)	265 (15.90)	224 (53.98)	110 (27.57)	117 (52.23)	
Treatments for diabetes, *n* (%)							0.400
No	4013 (96.16)	1461 (96.75)	1596(95.74)	398 (95.90)	380 (95.24)	218 (97.32)	
Yes	162 (3.84)	49 (3.25)	71 (4.26)	17 (4.10)	19 (4.76)	6 (2.68)	
Treatments for depression, *n* (%)							<0.001
No	4191 (99.43)	1507 (99.80)	1662 (99.70)	411 (99.04)	393 (98.50)	218 (97.32)	
Yes	24 (0.57)	3 (0.20)	5 (0.30)	4 (0.96)	6 (1.50)	6 (2.68)	
Cognition scores, Mean (SD)	11.13 (3.97)	12.52 (3.64)	10.95 (3.84)	9.31 (3.84)	9.96 (4.00)	8.71 (3.85)	<0.001
BMI (kg/m^2^), Mean (SD)	23.90 (4.05)	24.21 (3.94)	23.84 (3.87)	23.40 (4.22)	23.75 (4.98)	23.42 (3.73)	<0.001
SBP (mmHg), Mean (SD)	128.23 (20.06)	128.70 (19.31)	128.41 (20.49)	128.20 (20.92)	128.09 (19.84)	123.95 (20.15)	0.024
DBP (mmHg), Mean (SD)	75.58 (11.84)	76.30 (11.79)	75.35 (11.72)	75.28 (12.58)	75.73 (11.49)	72.71 (11.81)	<0.001
TC (mg/dL), Mean (SD)	193.66 (38.39)	190.84 (37.14)	195.26 (38.96)	192.42 (36.86)	196.38 (40.18)	198.17 (40.69)	0.002
HDL-C (mg/dL), Mean (SD)	51.12 (15.19)	49.58 (14.49)	51.65 (15.10)	53.02 (15.65)	51.71 (16.55)	52.91 (16.27)	<0.001
LDL-C (mg/dL), Mean (SD)	117.34 (34.87)	115.35 (33.92)	119.10 (36.27)	117.14 (33.32)	116.95 (35.65)	118.71 (31.44)	0.049
HbA1c (%), Mean (SD)	5.27 (0.76)	5.24 (0.74)	5.26 (0.70)	5.27 (0.73)	5.38 (1.04)	5.32 (0.74)	0.016
CRP (mg/L), Mean (SD)	2.47 (6.95)	2.63 (8.77)	2.49 (5.95)	1.87 (2.96)	2.56 (7.06)	2.27 (4.70)	0.381
FBG (mg/dL), Mean (SD)	108.75(31.97)	110.31(35.32)	107.73(27.68)	104.71(25.21)	112.45(41.01)	106.76(29.77)	0.001
TG (mg/dL), Mean (SD)	129.28(93.59)	132.71(98.19)	126.64(89.55)	117.37(70.53)	136.23(107.78)	135.46(99.45)	0.010

^a^ Including participants who were legally married or in a common-law relationship. ^b^ Including participants who were never married, separated, widowed, or divorced. Abbreviations: CVD, cardiovascular disease; BMI, body mass index; SBP, systolic blood pressure; DBP, diastolic blood pressure; TC, total cholesterol; HDL-C, high-density lipoprotein cholesterol; LDL-C, low-density lipoprotein cholesterol; HbA1c, glycosylated hemoglobin; CRP, C-reactive protein; FBG, fasting blood glucose; TG, triglyceride.

## Data Availability

The data used in this study are from CHARLS 2011-2018. The data are publicly available and can be downloaded from the CHARLS website: https://charls.pku.edu.cn/ (accessed on 5 March 2024).

## Supplementary Materials

The following supporting information can be downloaded at https://www.mdpi.com/article/10.3390/nu16244300/s1: Supplementary Table S1. The summary information on the model of trajectories of depressive symptoms. Supplementary Table S2. Collinearity diagnostic measures of the variables. Supplementary Table S3. Association between quartiles of TyG Index and trajectories of depressive symptoms in participants without treatments for diabetes or depression. Supplementary Table S4. Association between quartiles of TG/HDL-C ratio and trajectories of depressive symptoms in participants without treatments for diabetes or depression. Supplementary Table S5. Comparison of baseline characteristics between participants and those excluded because of failure to follow up. Supplementary Figure S1. Flowchart of sampling. Supplementary Figure S2. CESD-10 scores of participants with different depressive symptom trajectories across four waves. Supplementary Figure S3. Subgroup analysis of association between quartiles of TyG Index and TG/HDL-C ratio and trajectories of depressive symptoms by age (45–64 or ≥ 65 years) and sex (male or female). Supplementary Figure S4. Subgroup analysis of association between quartiles of TyG Index and TG/HDL-C ratio and trajectories of depressive symptoms by place of residence (rural or urban) and BMI (<24 or ≥24).

## References

[1] McCarron R.M., Shapiro B., Rawles J., Luo J. (2021). Depression. Ann. Intern. Med..

[2] Ferrari A.J., Santomauro D.F., Aali A., Abate Y.H., Abbafati C., Abbastabar H., Abd ElHafeez S., Abdelmasseh M., Abd-Elsalam S., Abdollahi A. (2024). Global incidence, prevalence, years lived with disability (YLDs), disability-adjusted life-years (DALYs), and healthy life expectancy (HALE) for 371 diseases and injuries in 204 countries and territories and 811 subnational locations, 1990–2021: A systematic analysis for the Global Burden of Disease Study 2021. Lancet.

[3] Cai H., Jin Y., Liu R., Zhang Q., Su Z., Ungvari G.S., Tang Y.L., Ng C.H., Li X.H., Xiang Y.T. (2023). Global prevalence of depression in older adults: A systematic review and meta-analysis of epidemiological surveys. Asian J. Psychiatry.

[4] Lu J., Xu X., Huang Y., Li T., Ma C., Xu G., Yin H., Xu X., Ma Y., Wang L. (2021). Prevalence of depressive disorders and treatment in China: A cross-sectional epidemiological study. Lancet Psychiatry.

[5] Fan X., Guo X., Ren Z., Li X., He M., Shi H., Zha S., Qiao S., Zhao H., Li Y. (2021). The prevalence of depressive symptoms and associated factors in middle-aged and elderly Chinese people. J. Affect. Disord..

[6] Nouwen A., Winkley K., Twisk J., Lloyd C.E., Peyrot M., Ismail K., Pouwer F. (2010). Type 2 diabetes mellitus as a risk factor for the onset of depression: A systematic review and meta-analysis. Diabetologia.

[7] Pashaki M.S., Mezel J.A., Mokhtari Z., Gheshlagh R.G., Hesabi P.S., Nematifard T., Khaki S. (2019). The prevalence of comorbid depression in patients with diabetes: A meta-analysis of observational studies. Diabetes Metab. Syndr..

[8] Moulton C.D., Pickup J.C., Ismail K. (2015). The link between depression and diabetes: The search for shared mechanisms. Lancet Diabetes Endocrinol..

[9] Krupa A.J., Dudek D., Siwek M. (2024). Consolidating evidence on the role of insulin resistance in major depressive disorder. Curr. Opin. Psychiatry.

[10] Watson K., Nasca C., Aasly L., McEwen B., Rasgon N. (2018). Insulin resistance, an unmasked culprit in depressive disorders: Promises for interventions. Neuropharmacology.

[11] Zou X.H., Sun L.H., Yang W., Li B.J., Cui R.J. (2020). Potential role of insulin on the pathogenesis of depression. Cell Prolif..

[12] Pearson S., Schmidt M., Patton G., Dwyer T., Blizzard L., Otahal P., Venn A. (2010). Depression and insulin resistance: Cross-sectional associations in young adults. Diabetes Care.

[13] Rhee S.J., Min S., Hong M., Lee H., Lee H.S., Kang D.H., Ahn Y.M. (2023). The association between insulin resistance and depressive symptoms—A national representative cross-sectional study. J. Psychosom. Res..

[14] Ford A.H., Flicker L., Hankey G.J., Yeap B.B., Chubb S.A., Golledge J., Almeida O.P. (2015). Insulin resistance and depressive symptoms in older men: The health in men study. Am. J. Geriatr. Psychiatry.

[15] Watson K.T., Simard J.F., Henderson V.W., Nutkiewicz L., Lamers F., Nasca C., Rasgon N., Penninx B. (2021). Incident Major Depressive Disorder Predicted by Three Measures of Insulin Resistance: A Dutch Cohort Study. Am. J. Psychiatry.

[16] Kan C., Silva N., Golden S.H., Rajala U., Timonen M., Stahl D., Ismail K. (2013). A systematic review and meta-analysis of the association between depression and insulin resistance. Diabetes Care.

[17] Fernandes B.S., Salagre E., Enduru N., Grande I., Vieta E., Zhao Z. (2022). Insulin resistance in depression: A large meta-analysis of metabolic parameters and variation. Neurosci. Biobehav. Rev..

[18] Singh B., Saxena A. (2010). Surrogate markers of insulin resistance: A review. World J. Diabetes.

[19] Guerrero-Romero F., Simental-Mendía L.E., González-Ortiz M., Martínez-Abundis E., Ramos-Zavala M.G., Hernández-González S.O., Jacques-Camarena O., Rodríguez-Morán M. (2010). The product of triglycerides and glucose, a simple measure of insulin sensitivity. Comparison with the euglycemic-hyperinsulinemic clamp. J. Clin. Endocrinol. Metab..

[20] Baneu P., Văcărescu C., Drăgan S.R., Cirin L., Lazăr-Höcher A.I., Cozgarea A., Faur-Grigori A.A., Crișan S., Gaiță D., Luca C.T. (2024). The Triglyceride/HDL Ratio as a Surrogate Biomarker for Insulin Resistance. Biomedicines.

[21] Shi Y.Y., Zheng R., Cai J.J., Qian S.Z. (2021). The association between triglyceride glucose index and depression: Data from NHANES 2005–2018. BMC Psychiatry.

[22] Zhang X., Zhao D., Guo S., Yang J., Liu Y. (2024). Association between triglyceride glucose index and depression in hypertensive population. J. Clin. Hypertens..

[23] Zhang S., Hou Z., Fei D., Zhang X., Gao C., Liu J., Jin M., Zhai X., Zhou Y., Ni A. (2023). Associations between triglyceride glucose index and depression in middle-aged and elderly adults: A cross-sectional study. Medicine.

[24] Jin M., Lv P., Liang H., Teng Z., Gao C., Zhang X., Ni A., Cui X., Meng N., Li L. (2023). Association of triglyceride-glucose index with major depressive disorder: A cross-sectional study. Medicine.

[25] Behnoush A.H., Mousavi A., Ghondaghsaz E., Shojaei S., Cannavo A., Khalaji A. (2024). The importance of assessing the triglyceride-glucose index (TyG) in patients with depression: A systematic review. Neurosci. Biobehav. Rev..

[26] Zheng L., Cui C., Yue S., Yan H., Zhang T., Ding M., Sun Q., He C., Ren H. (2023). Longitudinal association between triglyceride glucose index and depression progression in middle-aged and elder adults: A national retrospective cohort study. Nutr. Metab. Cardiovasc. Dis..

[27] Wei Y.G., Cai D.B., Liu J., Liu R.X., Wang S.B., Tang Y.Q., Zheng W., Wang F. (2020). Cholesterol and triglyceride levels in first-episode patients with major depressive disorder: A meta-analysis of case-control studies. J. Affect. Disord..

[28] Vancampfort D., Correll C.U., Wampers M., Sienaert P., Mitchell A.J., De Herdt A., Probst M., Scheewe T.W., De Hert M. (2014). Metabolic syndrome and metabolic abnormalities in patients with major depressive disorder: A meta-analysis of prevalences and moderating variables. Psychol. Med..

[29] Pan A., Keum N., Okereke O.I., Sun Q., Kivimaki M., Rubin R.R., Hu F.B. (2012). Bidirectional association between depression and metabolic syndrome: A systematic review and meta-analysis of epidemiological studies. Diabetes Care.

[30] Han A.L. (2022). Association between lipid ratio and depression: A cross-sectional study. Sci. Rep..

[31] Musliner K.L., Munk-Olsen T., Eaton W.W., Zandi P.P. (2016). Heterogeneity in long-term trajectories of depressive symptoms: Patterns, predictors and outcomes. J. Affect. Disord..

[32] Xiang X. (2020). Seven-Year Trajectories of Depressive Symptoms and Their Predictors Among Older Americans. J. Aging Health.

[33] Zhang B.Y., Lin Y.D., Hu M.J., Sun Y., Xu M.H., Hao J.J., Zhu C.R. (2022). Associations between trajectories of depressive symptoms and rate of cognitive decline among Chinese middle-aged and older adults: An 8-year longitudinal study. J. Psychosom. Res..

[34] Min J., Cao Z., Chen H., Wang X., Xu C. (2024). Trajectories of depressive symptoms and risk of cardiovascular disease, cancer and mortality: A prospective cohort study. Gen. Psychiatr..

[35] Saeed Mirza S., Ikram M.A., Freak-Poli R., Hofman A., Rizopoulos D., Tiemeier H. (2018). 12 Year Trajectories of Depressive Symptoms in Community-Dwelling Older Adults and the Subsequent Risk of Death Over 13 Years. J. Gerontol. A Biol. Sci. Med. Sci..

[36] Zhao Y., Hu Y., Smith J.P., Strauss J., Yang G. (2014). Cohort profile: The China Health and Retirement Longitudinal Study (CHARLS). Int. J. Epidemiol..

[37] Zhao Y., Strauss J., Yang G., Giles J., Hu P.P., Hu Y., Lei X., Liu M., Park A., Smith J.P. (2013). China Health and Retirement Longitudinal Study: 2011–2012 National Baseline User’s Guide.

[38] Chen X., Crimmins E., Hu P.P., Kim J.K., Meng Q., Strauss J., Wang Y., Zeng J., Zhang Y., Zhao Y. (2019). Venous Blood-Based Biomarkers in the China Health and Retirement Longitudinal Study: Rationale, Design, and Results From the 2015 Wave. Am. J. Epidemiol..

[39] Boey K.W. (1999). Cross-validation of a short form of the CES-D in Chinese elderly. Int. J. Geriatr. Psychiatry.

[40] Chen H., Mui A.C. (2014). Factorial validity of the Center for Epidemiologic Studies Depression Scale short form in older population in China. Int. Psychogeriatr..

[41] Andresen E.M., Malmgren J.A., Carter W.B., Patrick D.L. (1994). Screening for depression in well older adults: Evaluation of a short form of the CES-D (Center for Epidemiologic Studies Depression Scale). Am. J. Prev. Med..

[42] Zhao Y., Crimmins E., Hu P.P., Hu Y., Ge T., Kim J.K., Strauss J., Yang G., Yin X., Wang Y. Blood Data Users’ Guide. https://charls.pku.edu.cn/wenjian/xuejianshujuyonghushiyongshouce.pdf.

[43] Tahapary D.L., Pratisthita L.B., Fitri N.A., Marcella C., Wafa S., Kurniawan F., Rizka A., Tarigan T.J.E., Harbuwono D.S., Purnamasari D. (2022). Challenges in the diagnosis of insulin resistance: Focusing on the role of HOMA-IR and Tryglyceride/glucose index. Diabetes Metab. Syndr..

[44] Wang Y., Chen X., Shi J., Du M., Li S., Pang J., Qiao J., Zhao Y., Chen Q., Guo Y. (2024). Relationship between triglyceride-glucose index baselines and trajectories with incident cardiovascular diseases in the elderly population. Cardiovasc. Diabetol..

[45] Liu T., Zhang Q., Wang Y., Ma X., Zhang Q., Song M., Cao L., Shi H. (2022). Association between the TyG index and TG/HDL-C ratio as insulin resistance markers and the risk of colorectal cancer. BMC Cancer.

[46] Zhang S., Cao C., Han Y., Hu H., Zheng X. (2024). A nonlinear relationship between the triglycerides to high-density lipoprotein cholesterol ratio and stroke risk: An analysis based on data from the China Health and Retirement Longitudinal Study. Diabetol. Metab. Syndr..

[47] Li C., Liu J., Ju Y., Liu B., Zhang Y. (2023). Multiple trajectories of depressive symptoms among Chinese in middle and late life: Characterization and risk factors. Int. J. Soc. Psychiatry.

[48] Xie Y., Ma M., Wu W., Zhang Y., Zhang Y., Tan X. (2021). Factors associated with depressive symptoms among the elderly in China: Structural equation model. Int. Psychogeriatr..

[49] Chen R., Chen Q., Lu G., Zhang M., Zhang M., Yang H., Qi K., Yu H., Zheng M., He Q. (2023). Sleep duration and depressive symptoms in Chinese middle-aged and older adults: The moderating effects of grip strength. J. Affect. Disord..

[50] Lin S., Wu Y., He L., Fang Y. (2023). Prediction of depressive symptoms onset and long-term trajectories in home-based older adults using machine learning techniques. Aging Ment. Health.

[51] Pei J., Hu M., Lu Q., Zhou P., Shang Y., Zhang H., Yang X., Li Y. (2024). Identifying the subgroups of depression trajectories among the middle-aged and older Chinese individuals with chronic diseases: An 8-year follow-up study based on CHARLS. Front. Public Health.

[52] Ji Y., Feng Y., Wu S., Wu Y., Wang J., Zhao X., Liu Y. (2023). Longitudinal trajectories of depressive symptoms: The role of multimorbidity, mobility and subjective memory. BMC Geriatr..

[53] Tian F., Yang H., Pan J. (2022). Association between functional disability and long-term trajectories of depressive symptoms: Evidence from the China Health and Retirement Longitudinal Study. J. Affect. Disord..

[54] Xie Y., Ma M., Wang W. (2023). Trajectories of depressive symptoms and their predictors in Chinese older population: Growth Mixture model. BMC Geriatr..

[55] Luo H., Li J., Zhang Q., Cao P., Ren X., Fang A., Liao H., Liu L. (2018). Obesity and the onset of depressive symptoms among middle-aged and older adults in China: Evidence from the CHARLS. BMC Public Health.

[56] Sible I.J., Jang J.Y., Sultzer D.L., Nation D.A. (2022). Visit-To-Visit Blood Pressure Variability and Subthreshold Depressive Symptoms in Older Adults. Am. J. Geriatr. Psychiatry.

[57] Beydoun M.A., Beydoun H.A., Dore G.A., Fanelli-Kuczmarski M.T., Evans M.K., Zonderman A.B. (2015). Total serum cholesterol, atherogenic indices and their longitudinal association with depressive symptoms among US adults. Transl. Psychiatry.

[58] Diniz B.S., Fisher-Hoch S., McCormick J. (2018). The association between insulin resistance, metabolic variables, and depressive symptoms in Mexican-American elderly: A population-based study. Int. J. Geriatr. Psychiatry.

[59] Jones B.L., Nagin D.S. (2013). A Note on a Stata Plugin for Estimating Group-based Trajectory Models. Sociol. Methods Res..

[60] Nagin D.S., Tremblay R.E. (2001). Analyzing developmental trajectories of distinct but related behaviors: A group-based method. Psychol. Methods.

[61] Nagin D.S., Odgers C.L. (2010). Group-based trajectory modeling in clinical research. Annu. Rev. Clin. Psychol..

[62] Wang J., Wang Y., Chen S., Fu T., Sun G. (2024). Urban-rural differences in key factors of depressive symptoms among Chinese older adults based on random forest model. J. Affect. Disord..

[63] Yang Y., Zhang X., Zhang Y., Zhao J., Jia J., Liu H., Song S. (2024). Metformin treatment improves depressive symptoms associated with type 2 diabetes: A 24-week longitudinal study. J. Affect. Disord..

[64] Rashidian H., Subramaniapillai M., Park C., Lipsitz O., Zuckerman H., Cao B., Lee Y., Gill H., Rodrigues R.N., Di Vincenzo J.D. (2023). Changes in insulin resistance following antidepressant treatment mediate response in major depressive disorder. J. Psychopharmacol..

[65] Zhang X., Wang Y., Yang X., Li Y., Gui J., Mei Y., Liu H., Guo L.L., Li J., Lei Y. (2024). Obesity and lipid indices as predictors of depressive symptoms in middle-aged and elderly Chinese: Insights from a nationwide cohort study. BMC Psychiatry.

[66] Chourpiliadis C., Zeng Y., Lovik A., Wei D., Valdimarsdóttir U., Song H., Hammar N., Fang F. (2024). Metabolic Profile and Long-Term Risk of Depression, Anxiety, and Stress-Related Disorders. JAMA Netw. Open.

[67] Kurauti M.A., Soares G.M., Marmentini C., Bronczek G.A., Branco R.C.S., Boschero A.C. (2021). Insulin and aging. Vitam. Horm..

[68] Li M.W., Chi X.W., Wang Y., Setrerrahmane S., Xie W.W., Xu H.M. (2022). Trends in insulin resistance: Insights into mechanisms and therapeutic strategy. Signal Transduct. Target. Ther..

[69] Kitada M., Koya D. (2021). Autophagy in metabolic disease and ageing. Nat. Rev. Endocrinol..

[70] Wang K., Zhao Y., Nie J., Xu H., Yu C., Wang S. (2021). Higher HEI-2015 Score Is Associated with Reduced Risk of Depression: Result from NHANES 2005-2016. Nutrients.

[71] Kapogiannis D., Manolopoulos A., Mullins R., Avgerinos K., Delgado-Peraza F., Mustapic M., Nogueras-Ortiz C., Yao P.J., Pucha K.A., Brooks J. (2024). Brain responses to intermittent fasting and the healthy living diet in older adults. Cell Metab..

[72] Noetel M., Sanders T., Gallardo-Gómez D., Taylor P., Del Pozo Cruz B., van den Hoek D., Smith J.J., Mahoney J., Spathis J., Moresi M. (2024). Effect of exercise for depression: Systematic review and network meta-analysis of randomised controlled trials. BMJ.

[73] Li X., Zhang W., Zhang W., Tao K., Ni W., Wang K., Li Z., Liu Q., Lin J. (2020). Level of physical activity among middle-aged and older Chinese people: Evidence from the China health and retirement longitudinal study. BMC Public Health.

[74] Zhang W., Wang T., Wang A. (2022). Impact of physical activity intensity on longitudinal trajectories of cognitive function and depressive symptoms in middle-aged and older Chinese adults: Eight-year prospective study. J. Affect. Disord..

[75] Roy A., Singh P.K., Saha S., Das A., Naithani M. (2022). Impact of lipid ratio as an objective indicator of mental health status in Indian Individuals with Diabetes mellitus: An observational pilot study. J. Public Health.

[76] Krzymien J., Ladyzynski P. (2024). Insulin resistance: Risk factors, diagnostic approaches and mathematical models for clinical practice, epidemiological studies, and beyond. Biocybern. Biomed. Eng..

[77] Du T., Yuan G., Zhang M., Zhou X., Sun X., Yu X. (2014). Clinical usefulness of lipid ratios, visceral adiposity indicators, and the triglycerides and glucose index as risk markers of insulin resistance. Cardiovasc. Diabetol..

[78] Huang R., Cheng Z., Jin X., Yu X., Yu J., Guo Y., Zong L., Sheng J., Liu X., Wang S. (2022). Usefulness of four surrogate indexes of insulin resistance in middle-aged population in Hefei, China. Ann. Med..

[79] Yaribeygi H., Farrokhi F.R., Butler A.E., Sahebkar A. (2019). Insulin resistance: Review of the underlying molecular mechanisms. J. Cell Physiol..

[80] Mastrototaro L., Roden M. (2021). Insulin resistance and insulin sensitizing agents. Metabolism.

[81] Bhatt S., Nagappa A.N., Patil C.R. (2020). Role of oxidative stress in depression. Drug. Discov. Today.

[82] Cui L., Li S., Wang S., Wu X., Liu Y., Yu W., Wang Y., Tang Y., Xia M., Li B. (2024). Major depressive disorder: Hypothesis, mechanism, prevention and treatment. Signal Transduct. Target. Ther..

[83] Janssen J. (2022). New Insights into the Role of Insulin and Hypothalamic-Pituitary-Adrenal (HPA) Axis in the Metabolic Syndrome. Int. J. Mol. Sci..

[84] Yokoyama K., Yamada T., Mitani H., Yamada S., Pu S., Yamanashi T., Matsumura H., Nakagome K., Kaneko K. (2015). Relationship between hypothalamic-pituitary-adrenal axis dysregulation and insulin resistance in elderly patients with depression. Psychiatry Res..

[85] Saccaro L.F., Tassone M., Tozzi F., Rutigliano G. (2024). Proton magnetic resonance spectroscopy of N-acetyl aspartate in first depressive episode and chronic major depressive disorder: A systematic review and meta-analysis. J. Affect. Disord..

[86] Lee S., Joo Y.J., Kim R.Y., Hwang J., Lim S.M., Yoon S., Kim J. (2020). Obesity May Connect Insulin Resistance to Decreased Neuronal Viability in Human Diabetic Brain. Obesity.

[87] Kakoty V., Kc S., Kumari S., Yang C.H., Dubey S.K., Sahebkar A., Kesharwani P., Taliyan R. (2023). Brain insulin resistance linked Alzheimer’s and Parkinson’s disease pathology: An undying implication of epigenetic and autophagy modulation. Inflammopharmacology.

[88] Grillo C.A., Woodruff J.L., Macht V.A., Reagan L.P. (2019). Insulin resistance and hippocampal dysfunction: Disentangling peripheral and brain causes from consequences. Exp. Neurol..

[89] Gruber J., Hanssen R., Qubad M., Bouzouina A., Schack V., Sochor H., Schiweck C., Aichholzer M., Matura S., Slattery D.A. (2023). Impact of insulin and insulin resistance on brain dopamine signalling and reward processing—An underexplored mechanism in the pathophysiology of depression?. Neurosci. Biobehav. Rev..

[90] Herman F.J., Simkovic S., Pasinetti G.M. (2019). Neuroimmune nexus of depression and dementia: Shared mechanisms and therapeutic targets. Br. J. Pharmacol..

[91] Khambaty T., Stewart J.C., Muldoon M.F., Kamarck T.W. (2014). Depressive symptom clusters as predictors of 6-year increases in insulin resistance: Data from the Pittsburgh Healthy Heart Project. Psychosom. Med..

[92] Barca M.L., Persson K., Eldholm R., Benth J., Kersten H., Knapskog A.B., Saltvedt I., Selbaek G., Engedal K. (2017). Trajectories of depressive symptoms and their relationship to the progression of dementia. J. Affect. Disord..

[93] Kim E.Y., Kim S.H., Ha K., Lee H.J., Yoon D.H., Ahn Y.M. (2015). Depression trajectories and the association with metabolic adversities among the middle-aged adults. J. Affect. Disord..

[94] Zheng X.W., Jiang M.L., Ren X., Han L.Y., Shen S.W. (2023). Distinct depressive symptom trajectories are associated with incident diabetes among Chinese middle-aged and older adults: The China Health and Retirement Longitudinal Study. J. Psychosom. Res..

[95] He Y., Tong L., Guo F., Zhao S., Zhang J., Guo X., Tao Y., Lin X., Jin L. (2022). Depression status and insulin resistance in adults with obesity: A cross-sectional study. J. Psychosom. Res..

[96] Salk R.H., Hyde J.S., Abramson L.Y. (2017). Gender differences in depression in representative national samples: Meta-analyses of diagnoses and symptoms. Psychol. Bull..

[97] Gado M., Tsaousidou E., Bornstein S.R., Perakakis N. (2024). Sex-based differences in insulin resistance. J. Endocrinol..

[98] Slavich G.M., Sacher J. (2019). Stress, sex hormones, inflammation, and major depressive disorder: Extending Social Signal Transduction Theory of Depression to account for sex differences in mood disorders. Psychopharmacology.

[99] Xu J., Zhang H., Zhang T., Sun J., Shi Q., Liu J., Tian G., Zhang B., Wang H., Wu Q. (2022). The “jolly fat” for the middle-aged and older adults in China, was education level considered?. J. Affect. Disord..

[100] Yuan L., Xu Q., Gui J., Liu Y., Lin F., Zhao Z., Sun J. (2023). Decomposition and comparative analysis of differences in depressive symptoms between urban and rural older adults: Evidence from a national survey. Int. Psychogeriatr..

[101] Chen L., Chang L., Lin H., Tu J., Chen X., Han Y. (2024). Depressive disorder benefits of cities: Evidence from the China. J. Affect. Disord..

[102] Liu X., Li N., Liu C., Ren X., Liu D., Gao B., Liu Y. (2016). Urban-rural disparity in utilization of preventive care services in China. Medicine.

[103] Xu J., Wang J., King M., Liu R., Yu F., Xing J., Su L., Lu M. (2018). Rural-urban disparities in the utilization of mental health inpatient services in China: The role of health insurance. Int. J. Health. Econ. Manag..

[104] Fan L., Wang Z., Zhao Y., Ma Y. (2023). Urban-Rural Disparities in Knowledge, Use and Perceived Benefits of Nutrition Labels in China: Evidence from 10 Provinces. Nutrients.

[105] Gutch M., Kumar S., Razi S.M., Gupta K.K., Gupta A. (2015). Assessment of insulin sensitivity/resistance. Indian J. Endocrinol. Metab..

[106] Batis C., Mendez M.A., Sotres-Alvarez D., Gordon-Larsen P., Popkin B. (2014). Dietary pattern trajectories during 15 years of follow-up and HbA1c, insulin resistance and diabetes prevalence among Chinese adults. J. Epidemiol. Community Health.

[107] Pei Z., Zhang J., Qin W., Hu F., Zhao Y., Zhang X., Cong X., Liu C., Xu L. (2022). Association between Dietary Patterns and Depression in Chinese Older Adults: A Longitudinal Study Based on CLHLS. Nutrients.

