# Supplementary material for: Association of TyG Index and TG/HDL-C Ratio with Trajectories of Depressive Symptoms: Evidence from China Health and Retirement Longitudinal Study
Source: Nutrients. 2024 Dec 12;16(24):4300. doi: 10.3390/nu16244300 (PMC11676214; doi:10.3390/nu16244300)
Supplement: Supplementary file 1 [file nutrients-16-04300-s001.zip › nutrients-3325903-supplementary.pdf]

Supplementary Online Content

Supplementary Table S1. The summary information on the model of trajectories of depressive symptoms
Supplementary Table S2. Collinearity diagnostic measures of the variables
Supplementary Table S3. Association between quartiles of TyG index and trajectories of depressive symptoms in participants without treatments for diabetes or depression
Supplementary Table S4. Association between quartiles of TG/HDL-C ratio and trajectories of depressive symptoms in participants without treatments for diabetes or depression
Supplementary Table S5. Comparison of baseline characteristics between participants and those excluded because of failure to follow up
Supplementary Figure S1. Flowchart of sampling
Supplementary Figure S2. CESD-10 scores of participants with different depressive symptom trajectories across four waves
Supplementary Figure S3. Subgroup analysis of association between quartiles of TyG Index and TG/HDL-C ratio and trajectories of depressive symptoms by age (45-64 or ⩾ 65 years) and sex (male or female)
Supplementary Figure S4. Subgroup analysis of association between quartiles of TyG Index and TG/HDL-C ratio and trajectories of depressive symptoms by place of residence (rural or urban) and BMI (<24 or ⩾ 24)

Supplementary Table S1. The summary information on the model of trajectories of depressive symptoms.

Number of Groups	Polynomial	BIC (n=4215)	Proportion of Group (%)					APPA
			Group 1	Group 2	Group 3	Group 4	Group 5	
1	3	-52926.71	100					1
2	3 3	-50841.37	71.40	28.60				0.96/0.91
3	3 3 3	-50364.75	52.93	37.02	10.05			0.91/0.85/0.90
4	3 3 3 3	-50309.70	39.45	38.02	17.56	4.96		0.85/0.76/0.78/0.86
5	3 3 3 3 3	-50199.93	35.26	38.09	10.42	10.68	5.56	0.84/0.75/0.72/0.73/0.85
**5**	**3 2 2 3 2**	**-50190.72**	**35.94**	**37.92**	**10.51**	**10.23**	**5.40**	**0.84/0.75/0.72/0.74/0.86**

Abbreviation: BIC, Bayesian information criterion; APPA, the average posterior probability of assignment.

Supplementary Table S2. Collinearity diagnostic measures of the variables.

**Variable**	**VIF**	**Tolerance**
Age	1.420	0.705
Sex	2.320	0.430
Household income	1.020	0.976
Marital status	1.060	0.947
Educational level	1.600	0.626
Place of residence	1.120	0.894
Smoking status	1.860	0.537
Drinking status	1.430	0.700
Sleep duration	1.050	0.956
Health status	1.170	0.856
Disabilities	1.050	0.954
Hypertension	1.350	0.738
Dyslipidemia	1.170	0.858
CVD	1.130	0.887
Diabetes	1.220	0.818
Cognition scores	1.460	0.683
BMI	1.350	0.739
SBP	2.860	0.350
DBP	2.680	0.374
**TC**	**4.870**	**0.205**
HDL-C	1.830	0.546
LDL-C	3.940	0.254
HbA1c	1.230	0.815
CRP	1.020	0.985
Quartiles of TyG index	2.030	0.492

Abbreviation: VIF, the variance inflation; CVD, Cardiovascular disease; BMI, body mass index; SBP, Systolic blood pressure; DBP, Diastolic blood pressure; TC, Total cholesterol; HDL-C, High-density lipoprotein cholesterol; LDL-C, Low-density lipoprotein cholesterol; HbA1c, Glycosylated hemoglobin; CRP, C-reactive protein; TyG, triglyceride-glucose; CESD-10, the ten-item Center for Epidemiologic Studies Depression Scale.

Supplementary Table S3. Association between quartiles of TyG index and trajectories of depressive symptoms in participants without treatments for diabetes or depression [a].

	Stable low (n=1458)	Stable moderate (n=1591)	Decreasing (n=395)	Increasing (n=375)	Stable high (n=212)
**Model 1: Unadjusted**					
Quartile1	1.00 (Reference)	1.00 (Reference)	1.00 (Reference)	1.00 (Reference)	1.00 (Reference)
Quartile2	1.00 (Reference)	1.10 (0.90,1.35)	1.24 (0.93,1.67)	1.11 (0.80,1.53)	1.20 (0.80,1.81)
Quartile3	1.00 (Reference)	0.99 (0.81,1.20)	0.83 (0.61,1.14)	0.97 (0.70,1.35)	1.17 (0.78,1.76)
Quartile4	1.00 (Reference)	0.89 (0.73,1.09)	**0.62 (0.44,0.86)**	1.13 (0.82,1.55)	0.96 (0.63,1.46)
**Model 2: Adjusted for baseline age and sex**					
Quartile1	1.00 (Reference)	1.00 (Reference)	1.00 (Reference)	1.00 (Reference)	1.00 (Reference)
Quartile2	1.00 (Reference)	1.06 (0.86,1.30)	1.15 (0.85,1.15)	1.01 (0.73,1.41)	1.06 (0.70,1.61)
Quartile3	1.00 (Reference)	0.91 (0.74,1.11)	0.71 (0.52,0.98)	0.82 (0.59,1.14)	0.91 (0.61,1.38)
Quartile4	1.00 (Reference)	0.83 (0.68,1.02)	**0.54 (0.38,0.76)**	0.97 (0.70,1.34)	0.76 (0.49,1.17)
**Model 3: Adjusted for baseline demographics and baseline health behaviors**					
Quartile1	1.00 (Reference)	1.00 (Reference)	1.00 (Reference)	1.00 (Reference)	1.00 (Reference)
Quartile2	1.00 (Reference)	1.07 (0.87,1.31)	1.20 (0.88,1.64)	1.04 (0.74,1.46)	1.10 (0.71,1.69)
Quartile3	1.00 (Reference)	0.97 (0.79,1.20)	0.81 (0.58,1.13)	0.91 (0.65,1.28)	1.06 (0.69,1.63)
Quartile4	1.00 (Reference)	0.90 (0.73,1.11)	**0.65 (0.45,0.93)**	1.12 (0.80,1.55)	0.93 (0.60,1.46)
**Model 4: Adjusted for baseline demographics, baseline health behaviors, and baseline health conditions**					
Quartile1	1.00 (Reference)	1.00 (Reference)	1.00 (Reference)	1.00 (Reference)	1.00 (Reference)
Quartile2	1.00 (Reference)	1.06 (0.85,1.31)	1.13 (0.81,1.57)	1.00 (0.71,1.41)	1.01 (0.65,1.59)
Quartile3	1.00 (Reference)	0.96 (0.78,1.19)	0.75 (0.53,1.07)	0.87 (0.61,1.23)	1.00 (0.63,1.56)
Quartile4	1.00 (Reference)	0.92 (0.73,1.14)	**0.64 (0.44,0.93)**	1.10 (0.78,1.56)	0.98 (0.61,1.57)
**Model 5: Adjusted for baseline demographics, baseline health behaviors, baseline health conditions, and clinical laboratory test data**					
Quartile1	1.00 (Reference)	1.00 (Reference)	1.00 (Reference)	1.00 (Reference)	1.00 (Reference)
Quartile2	1.00 (Reference)	1.05 (0.84,1.31)	1.15 (0.82,1.62)	1.06 (0.74,1.51)	1.09 (0.68,1.73)
Quartile3	1.00 (Reference)	0.97 (0.77,1.22)	0.77 (0.53,1.11)	0.97 (0.67,1.41)	1.10 (0.68,1.79)
Quartile4	1.00 (Reference)	0.95 (0.74,1.23)	**0.62 (0.41,0.96)**	1.21 (0.81,1.81)	1.10 (0.63,1.91)

[a] Data was reported as odds ratio (95%CI) from multinomial logistic regression.

Demographic factors were age, sex, household income, marital status, educational level, and Residence. Health behaviors consisted of smoking status, drinking status, and hours of sleep. Baseline health conditions included health status, disabilities, hypertension, dyslipidemia, cardiovascular disease (CVD), diabetes mellitus, and cognition scores. Clinical testing information consisted of BMI, systolic blood pressure, diastolic blood pressure, low-density lipoprotein cholesterol (LDL-C), glycated hemoglobin (HbA1c), and C-reactive protein (CRP) and high-density lipoprotein cholesterol (HDL-C, only for analyzing the TyG index).

Supplementary Table S4. Association between quartiles of TG/HDL-C ratio and trajectories of depressive symptoms in participants without treatments for diabetes or depression（Odds Ratio, 95%CI）.

	Stable low (n=1458)	Stable moderate (n=1591)	Decreasing (n=395)	Increasing (n=375)	Stable high (n=212)
**Model 1: Unadjusted**					
Quartile 1	1.00 (Reference)	1.00 (Reference)	1.00 (Reference)	1.00 (Reference)	1.00 (Reference)
Quartile 2	1.00 (Reference)	0.90(0.73,1.10)	0.86(0.63,1.17)	0.95(0.69,1.31)	0.66(0.44,1.00)
Quartile 3	1.00 (Reference)	1.02(0.83,1.24)	1.04(0.76,1.41)	0.93(0.67,1.30)	0.94(0.64,1.39)
Quartile 4	1.00 (Reference)	**0.73(0.60,0.90)**	**0.58(0.42,0.81)**	0.90(0.66,1.24)	0.70(0.47,1.04)
**Model 2: Adjusted for baseline age and gender**					
Quartile 1	1.00 (Reference)	1.00 (Reference)	1.00 (Reference)	1.00 (Reference)	1.00 (Reference)
Quartile 2	1.00 (Reference)	0.87 (0.71,1.07)	0.82(0.60,1.12)	0.90(0.65,1.24)	0.60(0.39,0.92)
Quartile 3	1.00 (Reference)	0.98 (0.80,1.21)	0.98(0.72,1.33)	0.87(0.62,1.21)	0.85(0.57,1.26)
Quartile 4	1.00 (Reference)	**0.71(0.58,0.88)**	**0.55(0.39,0.76)**	0.84(0.61,1.16)	0.62(0.41,0.94)
**Model 3: Adjusted for baseline demographics and baseline health behaviors**					
Quartile 1	1.00 (Reference)	1.00 (Reference)	1.00 (Reference)	1.00 (Reference)	1.00 (Reference)
Quartile 2	1.00 (Reference)	0.92(0.75,1.13)	0.92(0.66,1.27)	0.97(0.70,1.36)	0.67(0.43,1.04)
Quartile 3	1.00 (Reference)	1.05(0.85,1.29)	1.12(0.81,1.55)	0.96(0.68,1.36)	0.98(0.65,1.49)
Quartile 4	1.00 (Reference)	**0.79(0.64,0.97)**	**0.68(0.48,0.97)**	1.00(0.72,1.39)	0.78(0.51,1.19)
**Model 4: Adjusted for baseline demographics, baseline health behaviors, and baseline health conditions**					
Quartile 1	1.00 (Reference)	1.00 (Reference)	1.00 (Reference)	1.00 (Reference)	1.00 (Reference)
Quartile 2	1.00 (Reference)	0.93(0.75,1.15)	0.91(0.64,1.27)	0.96(0.68,1.35)	0.65(0.41,1.02)
Quartile 3	1.00 (Reference)	1.06(0.85,1.32)	1.07(0.76,1.50)	0.94(0.66,1.34)	0.93(0.60,1.44)
Quartile 4	1.00 (Reference)	0.82(0.66,1.03)	0.70(0.49,1.02)	1.01(0.72,1.43)	0.86(0.55,1.36)
**Model 5: Adjusted for baseline demographics, baseline health behaviors, baseline health conditions, and clinical laboratory test data**					
Quartile 1	1.00 (Reference)	1.00 (Reference)	1.00 (Reference)	1.00 (Reference)	1.00 (Reference)
Quartile 2	1.00 (Reference)	0.92(0.74,1.14)	0.93(0.66,1.31)	0.98(0.70,1.38)	0.67(0.42,1.06)
Quartile 3	1.00 (Reference)	1.06(0.85,1.33)	1.11(0.78,1.58)	0.98(0.68,1.41)	0.99(0.63,1.55)
Quartile 4	1.00 (Reference)	0.85(0.68,1.07)	0.75(0.51,1.10)	1.05(0.73,1.51)	0.95(0.59,1.52)

*Data was reported as risk ratios (95%CI) from multinomial logistic regression. Demographic factors were age, gender, household income, marital status, educational level, and Residence. Health behaviors consisted of smoking, drinking status, and hours of sleep. Baseline health conditions included health status, disabilities, hypertension, dyslipidemia, cardiovascular disease (CVD), diabetes mellitus, and cognition scores. Clinical laboratory test data consisted of BMI, systolic blood pressure, diastolic blood pressure, low-density lipoprotein cholesterol (LDL-C), glycated hemoglobin (HbA1c), and C-reactive protein (CRP) and high-density lipoprotein cholesterol (HDL-C, only for analyzing the TyG index).

Supplementary Table S5. Comparison of baseline characteristics between participants and those excluded because of failure to follow up.

**Characteristics**	**Participants (n=4215)**	**Fail to follow-up (n=3574)**	***p* value**
Age (years), Mean (SD)	57.27 (7.80)	61.90 (10.21)	<0.001
Sex, n (%)			0.165
Male	2017 (47.85)	1654 (46.28)	
Female	2198 (52.15)	1920 (53.72)	
Household income (RMB), Median (IQR)	10800 (1440, 10800)	8600 (1200, 27430)	<0.001
Marital status, n (%)			<0.001
Married [a]	3890 (92.29)	2985 (83.52)	
Single [b]	325 (7.71)	589 (16.48)	
Educational level, n (%)			<0.001
Illiterate	889 (21.09)	1291 (36.12)	
Primary school and below	1816 (43.08)	1408 (39.40)	
Middle school and above	1510 (35.82)	875 (24.48)	
Place of residence, n (%)			0.055
Rural	2730 (64.77)	2240 (62.67)	
Urban	1485 (35.23)	1334 (37.33)	
Smoking status, n (%)			0.100
Never	2565 (60.85)	2136 (59.76)	
Former	354 (8.40)	350 (9.79)	
Current	1296 (30.75)	1088 (30.44)	
Drinking status, n (%)			0.001
Never	2741 (65.03)	2463 (68.89)	
<1 time/month	352 (8.35)	278 (7.78)	
≥1 time/month	1122 (26.62)	834 (23.34)	
Sleep duration (hours/night), Mean (SD)	6.43 (1.79)	6.26 (1.98)	<0.001
Health status, n (%)			<0.001
Good	1039 (24.65)	704 (19.70)	
Fair	2114 (50.15)	1659 (46.42)	
Poor	1062 (25.20)	1211(33.88)	
Disabilities, n (%)			<0.001
No	3631 (86.14)	2806 (78.51)	
Yes	584 (13.86)	768 (21.49)	
Hypertension, n (%)			0.027
No	3195 (75.80)	2631 (73.61)	
Yes	1020 (24.20)	943 (26.39)	
Dyslipidemia, n (%)			0.157
No	3793 (89.99)	3250 (90.93)	
Yes	422 (10.01)	324 (9.07)	
CVD, n (%)			0.629
No	3717 (88.19)	3139 (87.83)	
Yes	498 (11.81)	435 (12.17)	
Diabetes, n (%)			0.520
No	3969 (94.16)	3353 (93.82)	
Yes	246 (5.84)	221 (6.18)	
Somatic-psychiatric comorbidity, n (%)			<0.001
No	3472 (82.37)	2718 (76.05)	
Yes	743 (17.63)	856 (23.95)	
Treatments for diabetes, n (%)			0.201
No	4053 (96.16)	3416 (95.58)	
Yes	162 (3.84)	158 (4.42)	
Treatments for depression, n (%)			0.248
No	4191 (99.43)	3546 (99.22)	

**Characteristics**	**Participants (n=4215)**	**Fail to follow-up (n=3574)**	***p* value**
Yes	24 (0.57)	28 (0.78)	
Cognition scores, Mean (SD)	11.13 (3.97)	9.45 (4.44)	<0.001
BMI (kg/m^2), Mean (SD)	23.90 (4.05)	23.19 (3.84)	<0.001
SBP (mmHg), Mean (SD)	128.23 (20.06)	132.75 (22.33)	<0.001
DBP (mmHg), Mean (SD)	75.58 (11.84)	75.89 (12.25)	0.253
TC (mg/dl), Mean (SD	193.66 (38.39)	194.86 (38.29)	0.166
HDL-C (mg/dl), Mean (SD)	51.12 (15.19)	51.68 (15.29)	0.106
LDL-C (mg/dl), Mean (SD)	117.34 (34.87)	117.77 (35.22)	0.591
HbA1c (%), Mean (SD)	5.27 (0.76)	5.31 (0.88)	0.021
CRP (mg/l), Mean (SD)	2.47 (6.95)	3.15 (8.34)	<0.001
FBG (mg/dl), Mean (SD)	108.75 (31.97)	110.592(37.79)	0.020
TG (mg/dl), Mean (SD)	129.28 (93.59)	129.18 (92.17)	0.964
TyG index, Mean (SD)	8.66 (0.65)	8.68 (0.65)	0.320
TG/HDL-C ratio, Mean (SD)	3.13 (3.95)	3.09 (3.77)	0.684
CESD-10 scores, Mean (SD)	8.00 (6.13)	9.15 (6.59)	<0.001

[a] Including participants who were legally married or in a common-law relationship.

[b] Including participants who were never married, separated, widowed, or divorced.

Abbreviation: CVD, Cardiovascular disease; BMI, body mass index; SBP, Systolic blood pressure; DBP, Diastolic blood pressure; TC, Total cholesterol; HDL-C, High-density lipoprotein cholesterol; LDL-C, Low-density lipoprotein cholesterol; HbA1c, Glycosylated hemoglobin; CRP, C-reactive protein; FBG, fasting blood glucose; TG, triglycerides; TyG, triglyceride-glucose; TG/HDL-C, triglyceride to high-density lipoprotein cholesterol; CESD-10, the ten-item Center for Epidemiologic Studies Depression Scale.

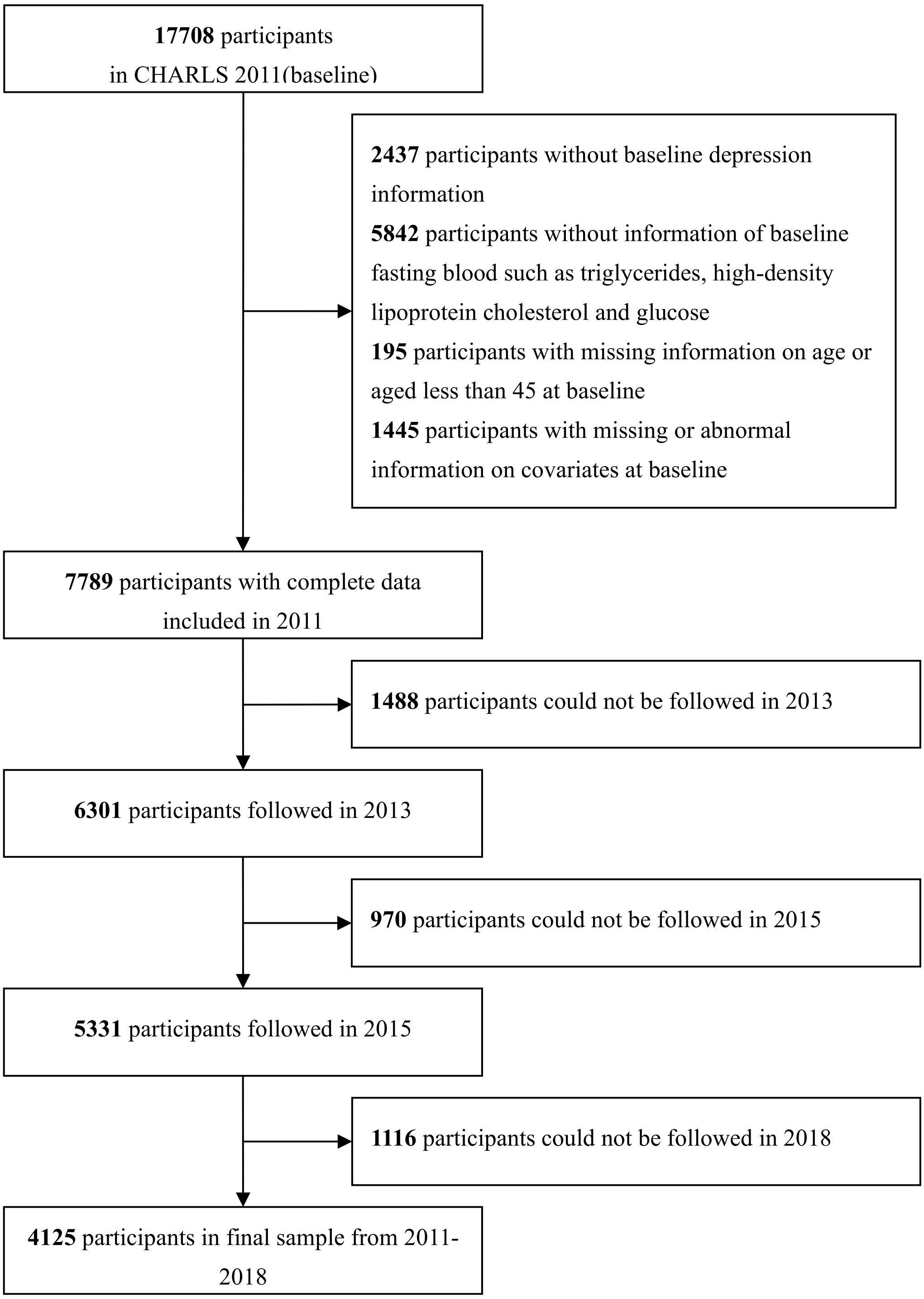

Supplementary Figure S1. Flowchart of sampling

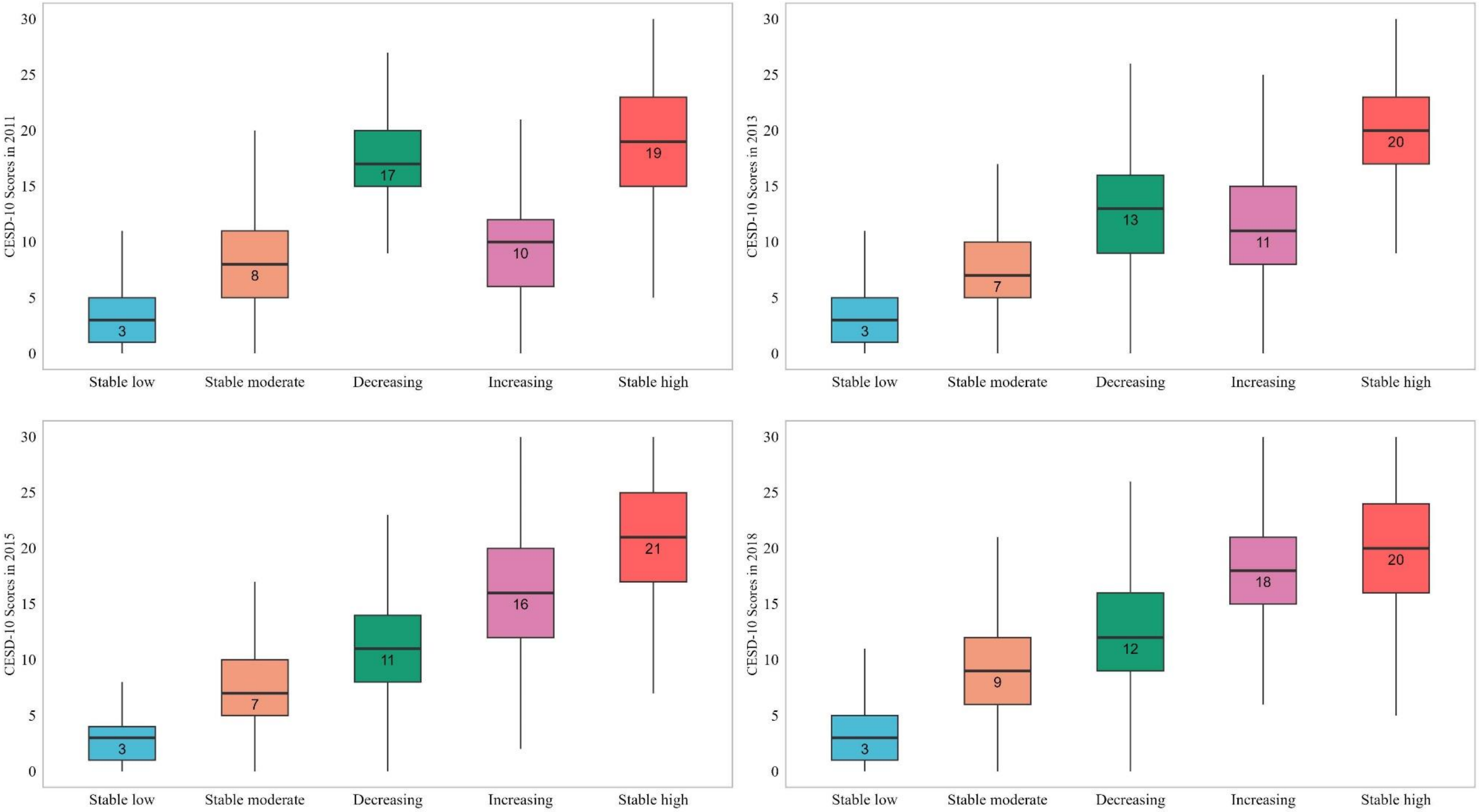

Supplementary Figure S2. CESD-10 scores of participants with different depressive symptom trajectories across four waves

Abbreviations: CESD-10, the ten-item Center for Epidemiologic Studies Depression Scale.

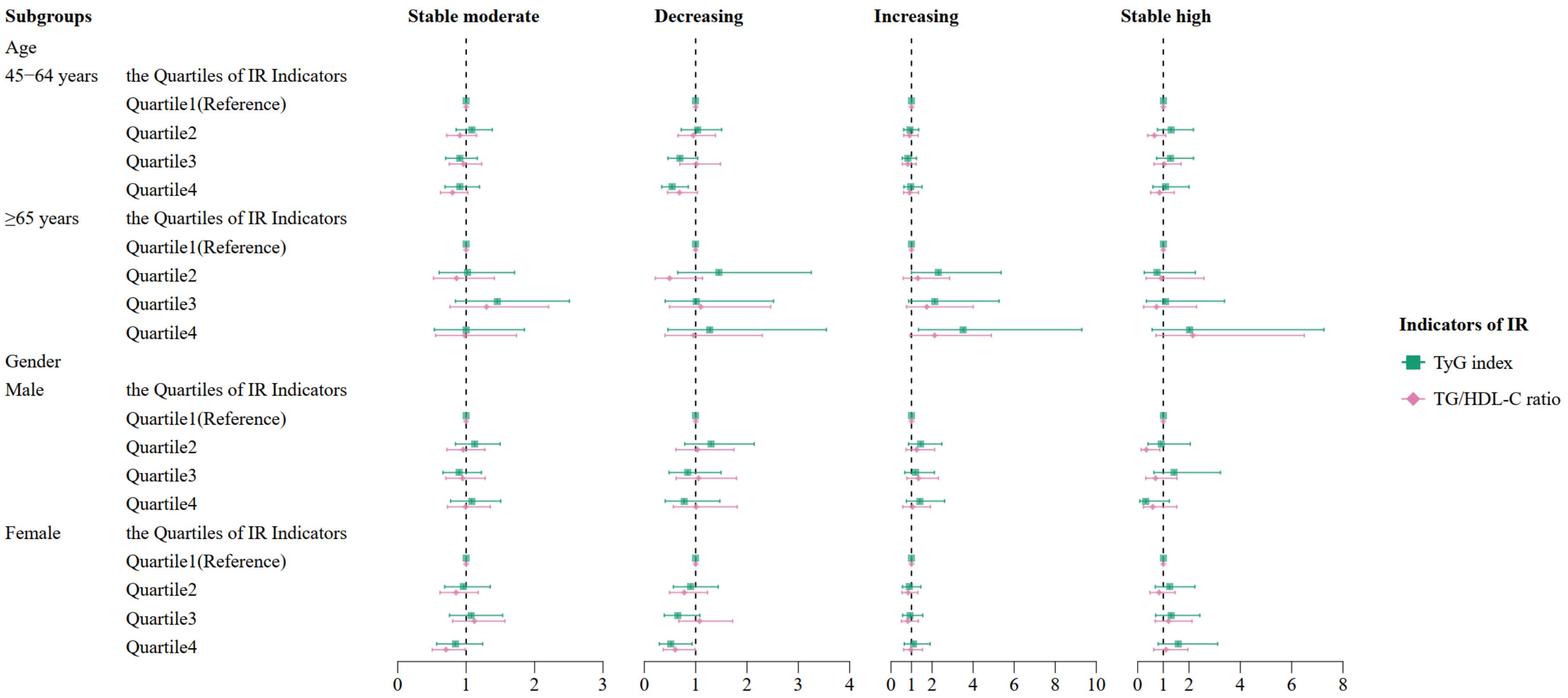

Supplementary Figure S3. Subgroup analysis of association between quartiles of TyG Index and TG/HDL-C ratio and trajectories of depressive symptoms by age (45-64 or ≥ 65 years) and sex (male or female)

Abbreviations: OR, odds ratio; CI, confidence intervals; TyG, triglyceride-glucose; TG/HDL-C, triglyceride to high-density lipoprotein cholesterol.

The multinomial logistic regression analysis was performed by using Model 5 (adjusted for baseline demographics, baseline health behaviors, baseline health conditions, and clinical laboratory test data).

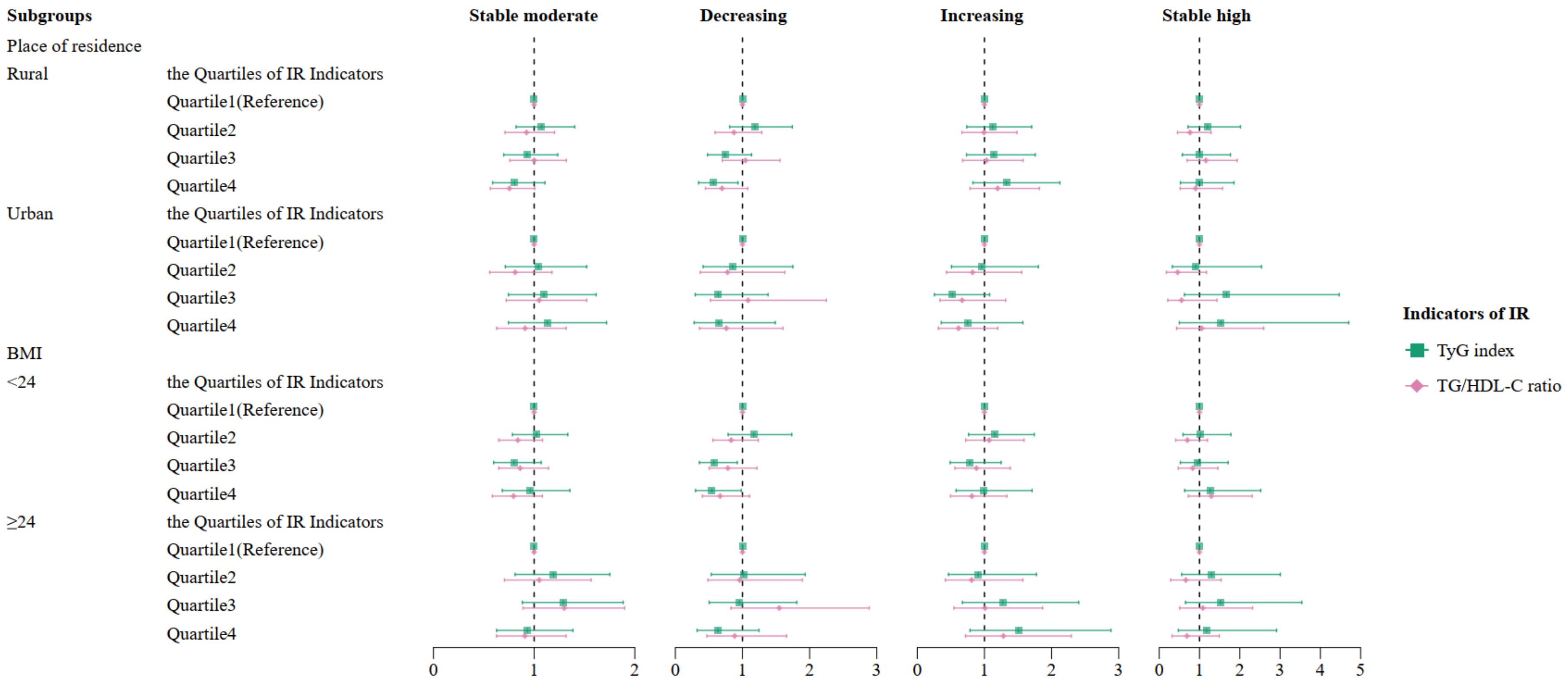

Supplementary Figure S4. Subgroup analysis of association between quartiles of TyG Index and TG/HDL-C ratio and trajectories of depressive symptoms by place of residence (rural or urban) and BMI (<24 or ≥ 24)

Abbreviations: OR, odds ratio; CI, confidence intervals; TyG, triglyceride-glucose; TG/HDL-C, triglyceride to high-density lipoprotein cholesterol.

The multinomial logistic regression analysis was performed by using Model 5 (adjusted for baseline demographics, baseline health behaviors, baseline health conditions, and clinical laboratory test data).
